# The dysregulation score method identifies epigenetic regulator genes that predict cancer prognosis and efficiency of cancer immunotherapy

**DOI:** 10.1016/j.omtn.2025.102781

**Published:** 2025-11-19

**Authors:** Jie Lyu, Hao Zhang, Jinjin Zhong, Zhen Feng

**Affiliations:** 1 College of Information and Engineering, The First Affiliated Hospital of Wenzhou Medical University, Wenzhou 325035, Zhejiang, People's Republic of China; 2 Wenzhou Key Laboratory of Biophysics, Wenzhou Institute, University of Chinese Academy of Sciences, Wenzhou 325001, Zhejiang, People's Republic of China; 3 Zhejiang Key Laboratory of Soft Matter Biomedical Materials, Wenzhou Institute, University of Chinese Academy of Sciences, Wenzhou 325000, Zhejiang, China

**Keywords:** MT: Bioinformatics, epigenetic regulator, cancer, prognosis, medicine target, cancer therapy

## Abstract

Epigenetic mechanisms play a crucial role in gene expression regulation during the initiation and progression of cancer. Despite this, over 600 epigenetic regulator (ER) genes, which are responsible for the reading, writing, and erasing of histone and DNA modifications, remain insufficiently characterized in the context of human cancer. In this study, we identified 272 cancer-specific ER genes that were dysregulated in cancer, as determined using a proposed dysregulation score method, based on analysis of over 19,000 paired tumor-normal human samples. Four novel dysregulated ER genes (DEGs), uniquely identified through this method, were shown to have roles in cell proliferation and invasion in melanoma cells. We proposed that loss-of-functional mutations within epigenetic domains may influence the dysregulation of ER genes. Signature scores derived from these DEGs can serve as convenient indicators of patient prognosis in different cancer types. Our findings demonstrated that DEGs in conjunction with immune checkpoints further enhance the prediction performance of the efficiency of cancer immunotherapy compared to using immune checkpoints alone, based on independent cancer cohorts. The DEG list is a valuable resource for translational cancer research, with implications for precision oncology and the development of more effective, individualized epigenetic medicines and therapy.

## Graphical abstract

**Figure:**
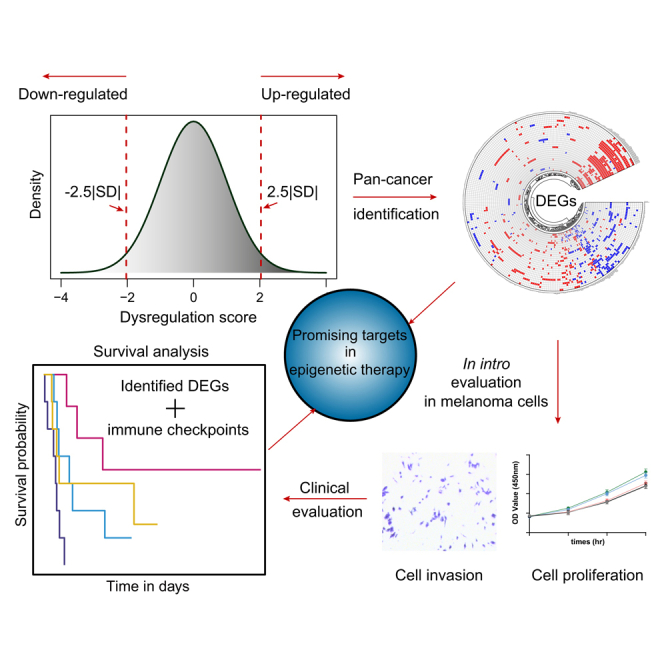

## Teaser

Lyu and colleagues identified 272 cancer-specific ER genes that were dysregulated in cancer, as determined using a proposed dysregulation score method. The dysregulated gene list is a valuable resource for cancer immunotherapy research, with implications for precision oncology and the development of more effective, individualized treatment strategies.

## Introduction

Epigenetics is the study that investigates protein factors and mechanisms that establish, maintain, or remove various covalent modification states on DNA sequences.^1^ The primary epigenetic modifications or processes include DNA methylation, histone modification, chromatin accessibility, and the regulation of three-dimensional chromatin structure. Epigenetic regulators (ERs) play a crucial role in adding, interpreting, removing, or remodeling chromatin states.^2^ ER genes are integral to genome evolution and tissue specificity.^3^ Chromatin regulators, which constitute a significant component of ERs, encompass a diverse array of well-characterized proteins.^4^ These proteins can be further categorized into several classes, including DNA methyltransferases (DNMTs), histone modifiers (methyltransferases, demethylases, acetyltransferases, and deacetylases), chromatin remodelers, and other related ERs.^5^

Genetic alterations represent a primary driving force in the initiation and progression of cancer.^6^ Nonetheless, the role of epigenetic modifications in cancer pathogenesis has garnered growing recognition, as these alterations can regulate gene expression without changing the underlying DNA sequence, potentially explaining tumor development in cancers with relatively few somatic mutations. Numerous ER genes are recurrently disrupted through genetic alterations in cancer, functioning as either oncogenes or tumor suppressor genes (TSGs) contingent upon the cellular context, thereby potentially causing perturbations in downstream gene expression.^7^^,^^8^ For instance, somatic mutations in ERs, including *TET2*, *DNMT3A*, and *ASXL1*, can act as initiating lesions that reprogram the epigenome, thereby priming cells for subsequent driver mutations and fostering oncogenic competence in hematologic malignancies.^9^ For example, extensive research has demonstrated that somatic mutations in chromatin remodeling complexes, especially the Brg/Brahma-associated factors complex, are present in over 20% of human cancers, underscoring their pivotal role in oncogenesis.^10^ Therefore, a large number of ERs may cooperate with canonical cancer genes to ignite malignant transformation.

Many ERs were already known as valid drug targets for pharmacological intervention in a variety of diseases, including autoimmune disorders and cancer.^11^^,^^12^ Notably, several pharmacological agents have been documented to function as histone deacetylase (HDAC) inhibitors, thereby reactivating TSGs.^13^ HDAC inhibitors, such as PCI-24781, have shown promising efficacy in suppressing cancer cell proliferation and metastasis.^14^ Furthermore, histone methyltransferases are integral to the epigenome, and the inhibitors targeting these enzymes are currently a focal point in cancer drug discovery efforts.^15^ Although traditional HDAC inhibitors have demonstrated efficacy in specific cancer treatments, they are often associated with significant off-target toxicities and limited effectiveness against solid tumors.^16^ These limitations have driven the development of next-generation HDAC inhibitors with improved selectivity, reduced toxicity, and enhanced therapeutic potential in solid malignancies. Consequently, there is a pressing need for a systematic compilation and characterization of pan-cancer-associated ER genes to facilitate the identification of more potential epigenetic targets for future anticancer therapies.

In prior research, cancer-associated ER genes have been identified within the context of tumor samples. For example, Cheng et al. examined the omics data from 24 cancer types within The Cancer Genome Atlas (TCGA) project. They discovered that ER expression signatures can stratify cancer patients into distinct prognostic groups, indicating either poor or favorable outcomes.^17^ Lu et al. differentiated between cancer-specific and non-cancer-specific ER genes by employing functional genomic data, which encompassed somatic mutations, cancer transcriptomics, and protein-protein interaction (PPI) network information.^18^ Additionally, a comprehensive analysis of ATP-dependent chromatin remodelers identified chromodomain-helicase-DNA-binding protein 7 as a gene target for treating colorectal cancer, highlighting the oncogenic roles of ERs in tumorigenesis.^19^

Immunotherapy has emerged as a transformative approach in the treatment of cancer, leveraging the body’s immune system to target and eliminate malignant cells. Immune checkpoint blockade (ICB), a major type of immunotherapy, works by targeting specific proteins on immune cells, such as cytotoxic T-lymphocyte antigen 4 (CTLA-4) and programmed cell death protein 1 (PD-1), which are known to inhibit immune responses. By blocking these checkpoints, ICB therapies effectively release the brakes on the immune system, allowing it to recognize and attack cancer cells more effectively. The approval of ipilimumab, a CTLA-4 inhibitor, marks a significant milestone in this field, underscoring the potential of ICB to improve patient outcomes across various cancer types. Despite its success, the heterogeneous and immunosuppressive tumor microenvironment (TME) frequently limits the efficacy of ICB therapy. ICB therapy is only effective in few cancer types, which can be attributed to several factors, including the presence of an immunosuppressive tumor TME and the metabolic adaptations of cancer cells that hinder effective immune responses. Consequently, investigators are seeking biomarkers beyond PD-1 and its ligand (PD-L1) to refine patient selection. The tumor immune dysfunction and exclusion (TIDE) computational framework integrates quantifiable features of the TME, such as the density, dysfunction, and spatial exclusion of tumor-infiltrating lymphocytes, to predict responsiveness to immune checkpoint blockade.^20^ In recent years, immunotherapy combined with epigenetic therapy has gradually emerged as a promising research area.^21^ Emerging studies have demonstrated a strong association between ER regulatory mechanisms and the effectiveness of immune checkpoint therapy. Specifically, DNMTs and HDACs, typically associated with transcriptional repression, modulate the expression of genes within immune checkpoint pathways, thereby contributing to improved immunotherapeutic outcomes.^22^ ERs can also potentiate T cell cytotoxicity or upregulate the expression of PD-1. When administered in combination with PD-1-blocking antibodies, this approach effectively suppresses tumor progression.^23^^,^^24^ Therefore, the combination of ERs with immune checkpoints represents an alternative strategy to help evaluate the efficiency of available cancer immunotherapeutic approaches.^25^ A systematic identification of nucleotide-based biomarkers, especially dysregulated ER genes (DEGs) that are capable of predicting the efficiency of the response to cancer immune therapy, is still needed. We recently identified hundreds of cancer immune-related ERs across 33 cancer types using the EPRIM algorithm, suggesting many ER genes are associated with immune regulation.^26^ When investigating the roles of somatic mutations on ER genes, we revealed the impact of mutated ER genes on molecular interaction networks, including ligand-receptor interactions and immune evasion.^27^ Our results offer novel insights into how somatic mutations in ER genes can affect tumor microenvironments and provide an algorithmic framework to enhance cancer immunotherapy strategies.^27^ However, developing a more efficient approach based solely on cancer transcriptomic data is still needed to uncover additional cancer immune-associated genes as novel therapeutic targets for cancer.

Until recently, the log2 fold-change (FC) ratio in tumor vs. normal samples was the most commonly used criterion for filtering differentially expressed genes (DEXGs) from the results of the differentially expressed gene identification algorithms like DESeq2, limma, and EdgeR.^28^^,^^29^^,^^30^ An FC threshold of 2 or 1.5 is commonly used as the cutoff to identify DEXGs. The FC method is a very intuitive method to identify DEXGs. DESeq2, edgeR, and other statistical tests, such as the Wilcoxon rank-sum test, relying on FC or *p* value, were also widely used previously, under the null hypothesis that no genes are differentially expressed.^31^ Since the threshold relying on *p* values may not be statistically sound and oversimplified in hypothesis, these methods may be less robust when applied to specific scenarios, for example, differentially expressed ER gene identification from a large number of cancer samples. Given the distinct expression pattern of ER genes, these traditional methods are likely to result in elevated false-positive rates and poor reproducibility in identifying DEGs. Notably, ER genes remain inadequately characterized at the omics level, and many of which may be missed in previous cancer gene predictions based on filtering by FC ratio. We anticipate that more DEGs in cancer can be identified from a larger number of cancer samples using a more robust differential gene identification method.

In this study, we conducted a cancer-type-specific DEG identification analysis from 690 compiled human ER genes based on a proposed dysregulation score, which can efficiently identify the differential ER gene expression patterns from hundreds of cancer and normal samples from the TCGA project. The successful identification of 272 cancer-specific DEGs, including dozens of novel DEGs, may lay the groundwork for elucidating the relationship between epigenetic regulation and cancer. Furthermore, our analysis highlights the therapeutic relevance of the identified DEGs based on cancer patient survival data. The DEGs demonstrate great potential as biomarkers for predicting patient prognosis and responsiveness to cancer immunotherapy, particularly immune checkpoint inhibitor (ICI) therapy. Mechanistically, DEGs may play key roles in modulating immune responses and orchestrating anti-tumor activity. Additionally, we identified candidate compounds capable of regulating DEG expression, offering promising avenues for therapeutic intervention.^12^ These findings collectively underscore the clinical value of DEG signatures in cancer immunotherapies, thereby enhancing the precision and efficacy of cancer treatment.

## Results

### The computational identification of DEGs based on a proposed dysregulation score

**Figure 1:**
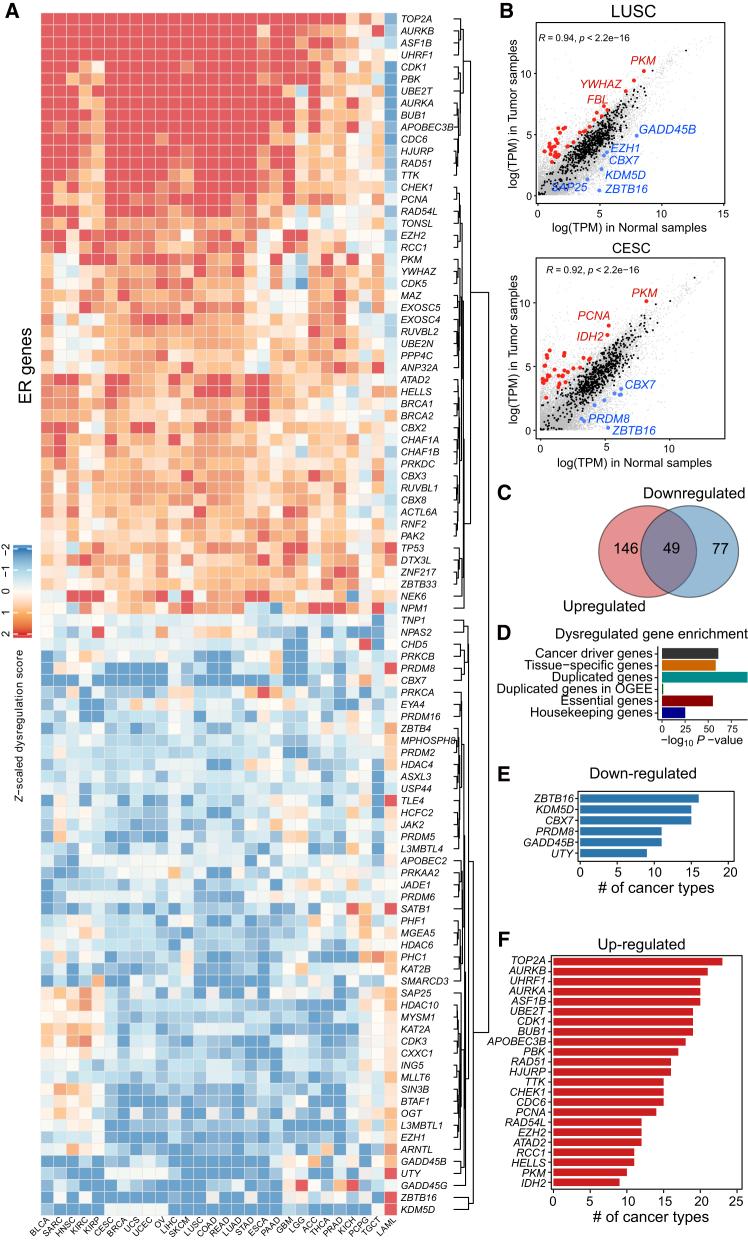
The DEG identification from human tumor and normal samples (A) Heatmap of dysregulation scores (DSs) of ERs in tumor and normal samples across 28 cancer types. Red color indicates upregulation (positive DS) in tumor samples, whereas blue color indicates downregulation (negative DS) in tumor samples compared with normal samples. The DSs in the heatmap are *Z*-scaled and centered. The top 50 upregulated and 50 downregulated genes are shown due to space limitations. (B) Scatterplots compare the log_2_TPM median expression of all genes in tumor and normal samples, for the two cancer types: lung squamous cell carcinoma (LUSC) and cervical squamous cell carcinoma and endocervical adenocarcinoma (CESC). ER genes are highlighted in black, with DEGs further highlighted in red for upregulated DEGs or in blue for downregulated DEGs. Non-ER genes are highlighted in gray. (C) A Venn diagram shows the overlap between upregulated genes and downregulated genes, with 49 genes as dual-role genes. (D) Enrichment of different functional gene sets in DEGs, with whole ER genes as background genes. *P* values from one-sided Fisher’s exact test are shown. (E) Barplot indicates the number of cancer types for the top-ranking associated downregulated ER genes. (F) Barplot indicates the number of cancer types for the top-ranking associated upregulated ER genes. Only ER genes that are dysregulated in more than nine cancer types are shown in (E) and (F). DEGs, dysregulated ER genes.

We carried out a systematic analysis on the ER expression pattern of 690 compiled human ER genes (Table S1) based on the pan-cancer tumor-normal samples from 28 cancer types.^32^ First, we calculated the log_2_FC of gene expression in each cancer type with normal samples as control, and found a large number of non-ER genes (gray dots) can be differentially expressed in different cancer types (Figures S1and S2). In contrast, the majority of the ER genes (black dots) are not differentially expressed (Figures S1 and S2). Therefore, we employed the dysregulation score (DS) metric, which accounts for the overall discrepancy of the paired tumor/normal data, as was used in a previous study, to identify ERs that were significantly dysregulated in a specific cancer type (Figure 1A).^33^ For specific cancer types, we found that the upregulated and downregulated ER genes were relatively few (Figure 1B), suggesting the DS score was a conservative score compared with the log_2_FC method. Of the DEGs in all cancer types, 146 DEGs were upregulated and 77 were downregulated (Figure 1C). In addition, 49 DEGs were dual-role genes that can be upregulated in specific cancer types but can be downregulated in other cancer types (Figure 1C). Among the 72 tissue-specific ER genes that were previously identified in humans, 19 genes overlapped with the DEGs (Fisher’s exact test, *p* < 0.001), suggesting the relationship between tissue evolution and cancer (Figure 1D).^3^ The DEGs were also associated with the cancer driver genes predicted by DORGE (Fisher’s exact test, *p* < 0.001), the duplicated genes (*p* < 0.001), essential genes (*p* < 0.001) and housekeeping genes (*p* < 0.001), but not with the compiled duplicated genes from the OGEE database (*p* = 6.07 × 10^−2^) (Figure 1D).

**Figure 2:**
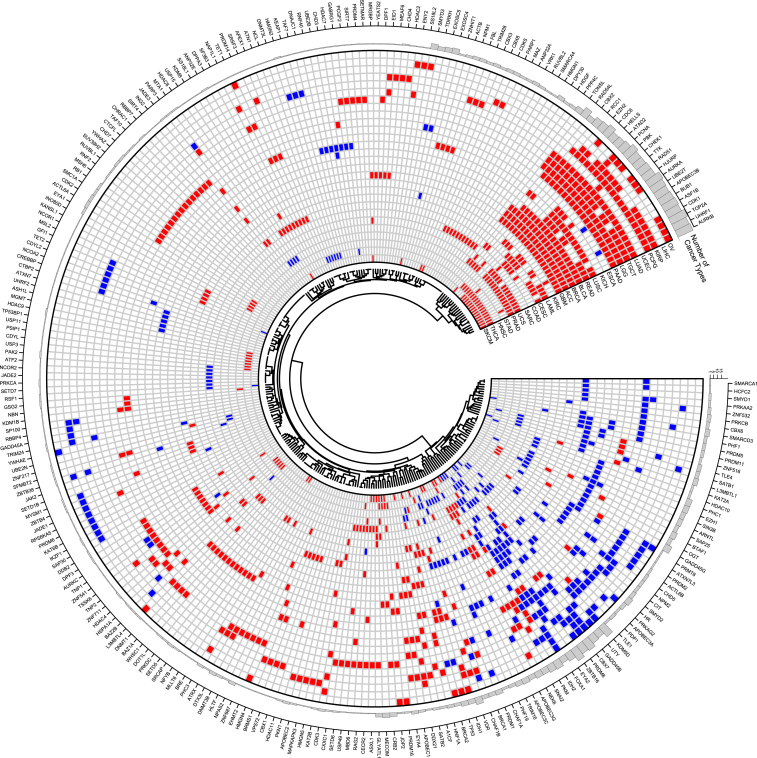
Circos plot showing the DEGs in 28 cancer types Red rectangle, upregulated ER genes; blue rectangle, downregulated ER genes. The circos plot was generated by the circlize R package (version 0.4.15).

DS score detected 106 upregulated ER genes as well as 71 downregulated ER genes in at least two cancer types (Figures 1E and 1F), where the most recurrently upregulated ER gene in the TCGA cancer types was *TOP2A* (Figure 1F). The depletion of *TOP2A* can suppress cell proliferation and induce apoptosis.^34^ We also observed that the most frequently downregulated ER was *ZBTB16* (Figure 1E). A recent study also indicated *ZBTB16* played a tumor suppressor role in cancer.^35^ In summary, a total of 272 DEGs were detected from at least one cancer type based on DS score, involving 1,081 cancer-specific DEG-cancer associations (Figure 2; Table S2).

We then characterized the identified DEGs separately for different epigenetic substrates (Figure S3). Interestingly, most of the histone H3K4/9/36 methylation erasers had a similar expression pattern across different cancer types (Figure S3). By contrast, we still found many DEGs with the same epigenetic substrates had distinct expression patterns. For example, *KDM5D* showed a quite different expression pattern compared with *KMT5A/B/C*. Interestingly, *KDM5A/B/C* were all reported to induce acute myeloid leukemia (LAML), while *KDM5D* has not been reported in literature to relate to LAML yet.^36^ Further efforts might be needed to explore the functional role of *KDM5D* in leukemia. Altogether, we suggested that the DEGs sharing the same substrates, which were previously thought to exhibit redundant biochemical activities, may have distinct functional roles in specific cancer types.

**Figure 3:**
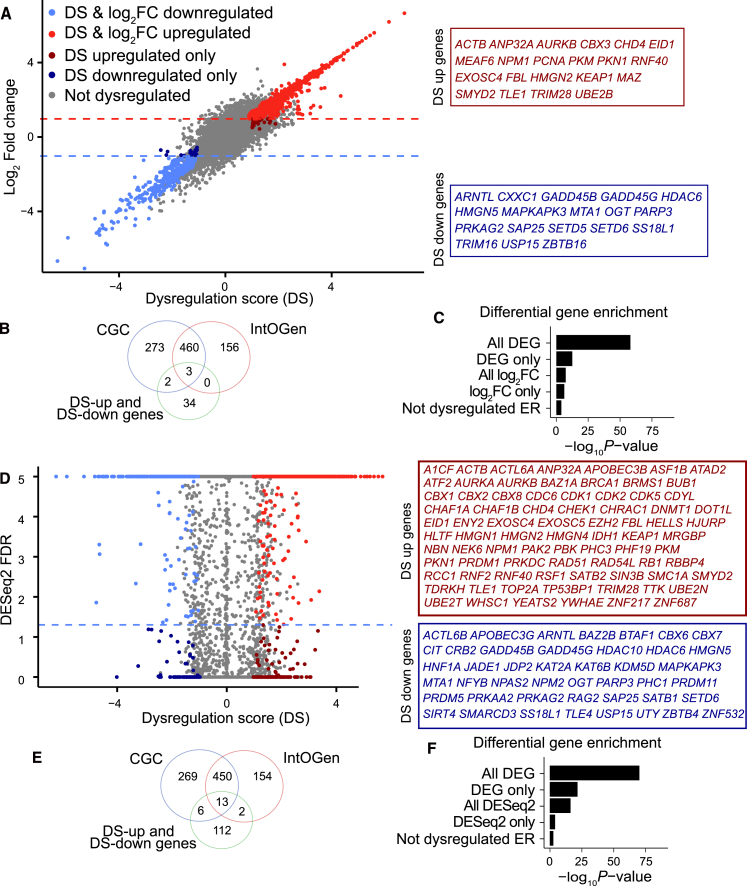
Dysregulation score identifies novel epigenetic driver genes (A) The scatterplot between dysregulation score and the log_2_FC method indicates novel cancer driver ER genes overlooked by known cancer gene databases. (B) Venn diagram illustrating that most of the ER genes that can be identified only by the DS method but not the log_2_FC method are not included in the CGC and IntOGen databases. (C) Enrichment of different DEG sets (*n* = 272 for all gene sets) identified from the DS method and the log_2_FC method in CGC cancer genes, with non-dysregulated ER genes as background genes. One-sided Fisher’s exact test is used to calculate the *p* value. (D) The scatterplot between dysregulation score and FDR from the DESeq2 method indicates novel cancer driver ER genes overlooked by known cancer gene databases. (E) Venn diagram illustrating that most of the ER genes that only can be identified by the DS method but not the DESeq2 are not included in the CGC and IntOGen databases. (F) Enrichment of different DEG sets identified from the DS method and the DESeq2 method in CGC cancer genes, with non-dysregulated ER genes as background genes. One-sided Fisher’s exact test is used to calculate the *p* value. CGC, Cancer Gene Census; IntOGen, Integrative Onco Genomics.

The proposed dysregulation score method can identify unique ER genes that are differentially expressed in cancer. A scatterplot between log_2_FC score and dysregulation score for ER genes indicated that DS score could identify 39 DEGs, but not by the log_2_FC method (Figure 3A; Table S3). We have shown the 39 unique genes that can only be identified by DS score in Figure 3A, including many well-known cancer driver genes (e.g., *CBX3* and *ZBTB16*), as well as uncertain cancer genes (e.g., *GADD45B*). Only a small proportion of these DEGs overlapped with known cancer driver genes in Cancer Gene Census (CGC) and Integrative Onco Genomics (IntOGen) databases, suggesting they may be genuine cancer driver genes (Figure 3B). In contrast, the log_2_FC method identified a large number of dysregulated genes (gray dots), many of which could represent data artifacts. We then performed a gene enrichment of the differentially expressed ER genes identified by different methods on cancer driver genes in the CGC database (Figure 3C). To enable a fair comparison, the differentially expressed ER genes identified by the log_2_FC method were downsampled so that the number of ER genes identified by log_2_FC, the number identified exclusively by log_2_FC, the total set of DEGs, and the non-dysregulated ER genes were equal (*n* = 272). Our analysis revealed that genes identified using the log_2_FC method showed a lower enrichment in known cancer driver genes, as documented in the CGC database, compared to those prioritized by the proposed DS method (Figure 3C).

### Preliminary functional validation of four DEGs identified by dysregulation score

**Figure 4:**
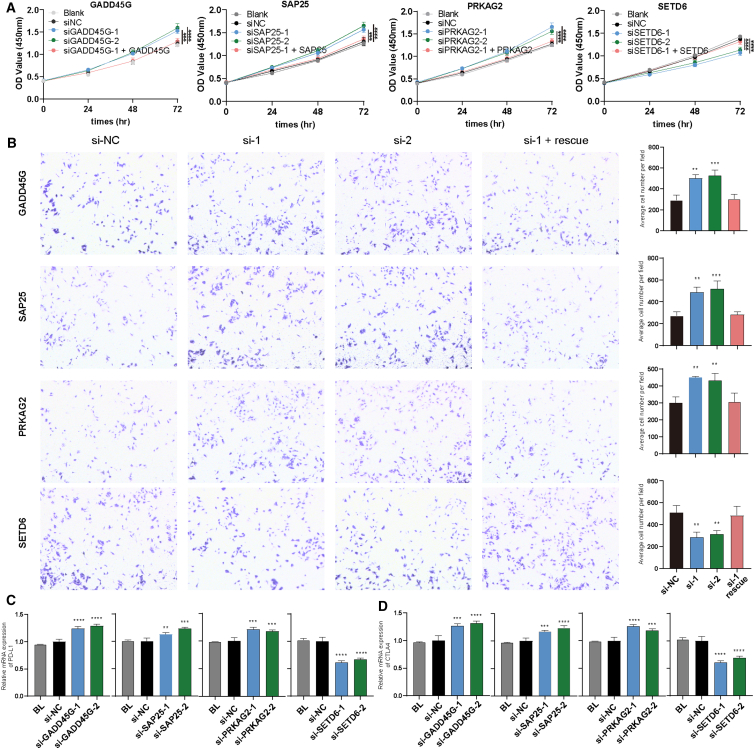
Functional investigation of four candidate cER genes in SK-MEL-2 cells (A) Four randomly chosen dysregulated cER genes were investigated in SK-MEL-2 cell line (*n* = 4) using CCK-8 assays. (B) Role of the candidate cER genes in cell invasion of SK-MEL-2 cell lines (*n* = 3). (C) The PD-L1 mRNA expression of four candidate cancer genes in SK-MEL-2 cell lines, as determined by qRT-PCR (*n* = 3). (D) The CTLA4 mRNA expression of four candidate cancer genes in SK-MEL-2 cell lines, as determined by qRT-PCR (*n* = 3). Statistical analysis was performed between si-NC and si-Genes. *P* values are calculated by an unpaired *t* test and indicated by star symbols, ∗*p* < 0.05; ∗∗*p* < 0.01; ∗∗∗*p* < 0.001; ∗∗∗∗*p* < 0.0001.

To validate the DS metric as a proof-of-concept, we selected four candidate DEGs (*GADD45G*, *SAP25*, *PRKAG2*, and *SETD6*) that were uniquely identified by the DS method. These genes were chosen based on a preliminary literature review, which indicated they had not been previously confirmed as cancer driver genes. All four genes exhibited high gene expression levels in SK-MEL-2 and A549 cell lines (representative of melanoma and non-small cell lung cancer, respectively). To functionally characterize the roles of these genes in cancer, we employed two independent small interfering RNAs (siRNAs) per gene to silence their expression in both cell lines. Forty-eight hours post-transfection, siRNA treatment achieved approximately 50% reduction in mRNA levels, as confirmed by quantitative reverse-transcription PCR (qRT-PCR) (Figure S4). Functional assessment using Cell Counting Kit-8 (CCK-8) proliferation assays revealed that siRNA-mediated knockdown of *GADD45G*, *SAP25*, and *PRKAG2* significantly increased cell proliferation compared with non-targeting control (NC) transfections, suggesting their potential roles as TSGs (Figures 4A and S5A). In contrast, silencing *SETD6* significantly reduced cell proliferation, implicating it as a candidate oncogene (Figures 4A and S5A). Furthermore, in the SK-MEL-2 cells, the downregulation of *GADD45G*, *SAP25*, and *PRKAG2* markedly promoted cell invasion and the downregulation of *SETD6* markedly inhibited cell invasion, as demonstrated by transwell assays, relative to negative control transfections (Figure 4B). Moreover, the knockdown of the four candidate genes significantly promoted or inhibited the well-known immune evasion markers, which included PD-L1 and CTLA4 at the mRNA level (Figures 4C, 4D, S5B, and S5C), suggesting the immune-regulatory roles of these candidate genes. The CCK-8 and transwell assays supported the utility of the DS metric in identifying novel cancer-associated ER genes that were overlooked previously. Further studies are warranted to elucidate the pathogenic mechanism underlying the roles of these DEGs in oncogenesis.

### DEGs can be regulated by somatic mutations in epigenetic domains

**Figure 5:**
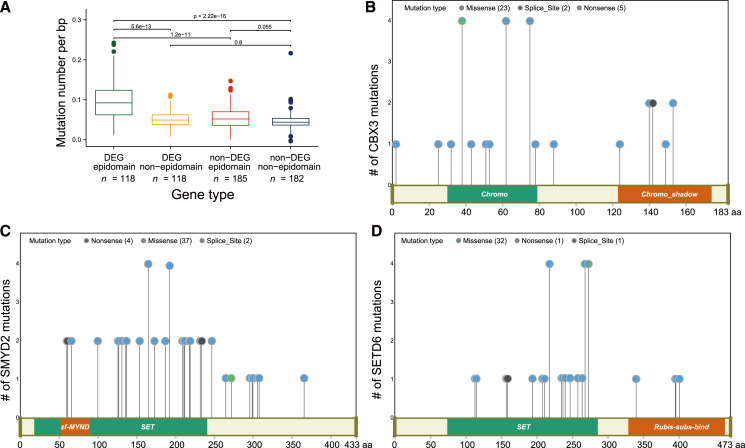
Somatic mutations are enriched in epigenetic domains of DEGs (A) Somatic mutation load (mutation number per basepair for ER and non-ER genes) is calculated for epigenetic domains in DEGs, non-epigenetic domains in DEGs, epigenetic domains in non-DEGs, and non-epigenetic domains in non-DEGs. *p* values are calculated for the indicated gene categories by two-sided Wilcoxon rank-sum test. Shown are *p* values adjusted by false discovery rate. (B)–(D) Lollipop plots showing somatic mutations in representative DEGs, including *CBX3*, *SMYD2*, and *SETD6*. The mutations are more enriched in epigenetic domains, compared with non-domain regions. DEGs, dysregulated ER genes.

We conducted additional analyses to determine whether DEGs could be modulated by somatic mutations. Specifically, we examined the somatic mutation burden within and outside major epigenetic domains independently (Ank, ARID, ASF1_hist_chap, BAH, Bromodomain, Chromo, DNA_methylase, Hist_deacetyl, JmjC, JmjC_2, JmjN, MBD, MBT, MOZ_SAS, Myb_DNA-binding, PHD, PWWP, RRM_1, SAM_1, SET, SIR2, SWIRM, TUDOR, UCH, WD40, zf-C2HC, zf-C2H2, zf-C3HC4, zf-CW, zf-CXXC, and zf-MYND domains in the pfam database). Overall, epigenetic domains in DEGs exhibited a greater somatic mutation burden compared with epigenetic domains in non-DEGs, as well as non-epigenetic domains in both DEGs and non-DEGs (Figure 5A). In non-DEGs, the epigenetic and non-epigenetic domains exhibited no significant differences (two-sided Wilcoxon rank-sum test, *p* = 0.055). This finding suggested that the observed distinctions of mutational frequencies between epigenetic and non-epigenetic domains are specific to DEGs. We have chosen three DEGs that contain Chromo or SET domains, two representative epigenetic domains, to examine the distribution of somatic mutations within vs. outside these domains and confirmed the trend in Figure 5A (Figures 5B–5D). Only three DEGs with higher mutational frequencies were shown here. Our analysis revealed that a higher number of somatic mutations were situated within the classical epigenetic domains for DEGs. This observation implied a potential link between somatic mutations in epigenetic domains and epigenetic regulation. Taken together, somatic mutations may alter protein conformation and the subsequent function of DEGs by disrupting epigenetic domains.

### DEGs are loosely connected in PPI networks and may play regulatory role via hub genes

**Figure 6:**
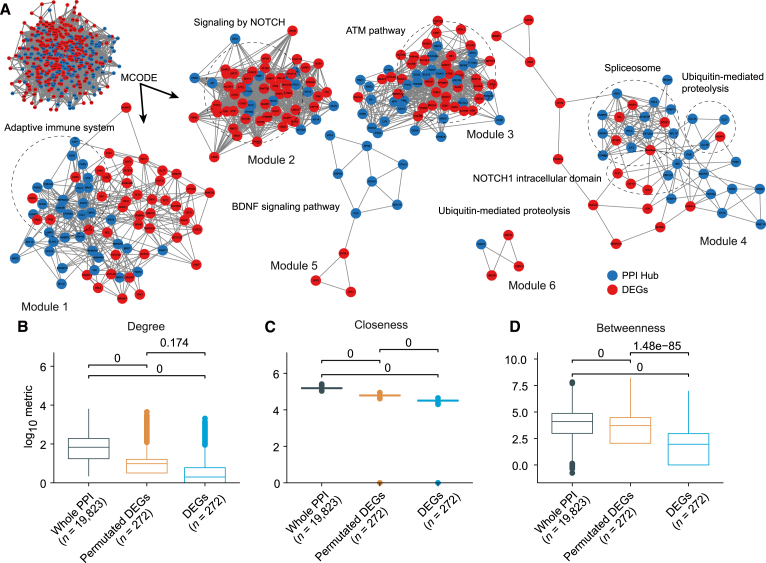
DEGs are loosely connected in PPI network (A) Module detection based on molecular complex detection algorithm for DEGs or hubs genes in PPI network. The PPI network was retrieved from the BioGRID database. The hub genes are defined as genes with degrees higher than the top 1% in the PPI network. Boxplots showing (B) degree, (C) closeness centrality, and (D) betweenness centrality metrics for all of the genes in the whole BioGRID PPI network, DEGs with permutated subnetwork structure, and DEGs in the BioGRID PPI network. *p* values are calculated for the indicated gene categories by the two-sided Wilcoxon rank-sum test. Genes from overrepresented functional terms are highlighted with dashed circles. DEGs, dysregulated ER genes.

Previous research has demonstrated that hub genes, defined as the set of genes exhibiting the highest connectivity within a PPI network, are often enriched with essential genes.^37^ Typically, the deletion of hub genes is detrimental to cellular function. To further investigate the DEGs within the PPI network, we obtained the complete BioGRID PPI network dataset and analyzed the degree of connectivity of the identified DEGs within this network. Of the 272 DEGs, 22 DEGs were actually hub genes in the analyzed PPI network. In contrast to the whole PPI network (the top left network in Figure 6A), the DEGs exhibited unexpectedly lower degrees, between centrality and closeness centrality compared with those in the whole network (Figures 6B–6D). We speculated that DEGs may indirectly affect the major structure of the PPI network through many possible mechanisms including small RNA sponges and epigenetic modification regulation. Notably, the DEGs exhibited even lower connection in PPI than that in the permutated ER subnetwork (Figures 6B–6D).

To further characterize the DEGs in the PPI network, we applied the molecular complex detection (MCODE) algorithm to detect network modules (Figure 6A) from the subnetwork including DEGs and hub non-ER genes. As a result, six gene modules were detected by the MCODE algorithm. The DEGs in the largest three modules (modules 1, 2, and 3) were densely connected, whereas the DEGs in the other three modules were loosely connected (Figure 6A). We also observed that the DEGs and hub genes were densely connected to each other but were sparsely connected between each other in modules 1, 4, and 5, suggesting DEGs may interact with hub genes to perform downstream regulatory activities. Gene ontology overrepresentation analysis revealed that specific hub genes in each module may be enriched in specific cancer-related biological processes, such as the ATM pathway and the adaptive immune system, prompting us to further characterize the cancer immunology of DEGs. Overall, we observed an unexpectedly low topological connectivity with other genes in the whole PPI network for DEGs (Figure 6), which can be explained by the fact that these ERs may regulate downstream targets through interacting with the hub genes in the PPI network. However, this explanation still requires support from more robust evidence.

### DEG-based gene signatures can classify molecular subtypes and help distinguish cancer patients with distinct survival outcomes

**Figure 7:**
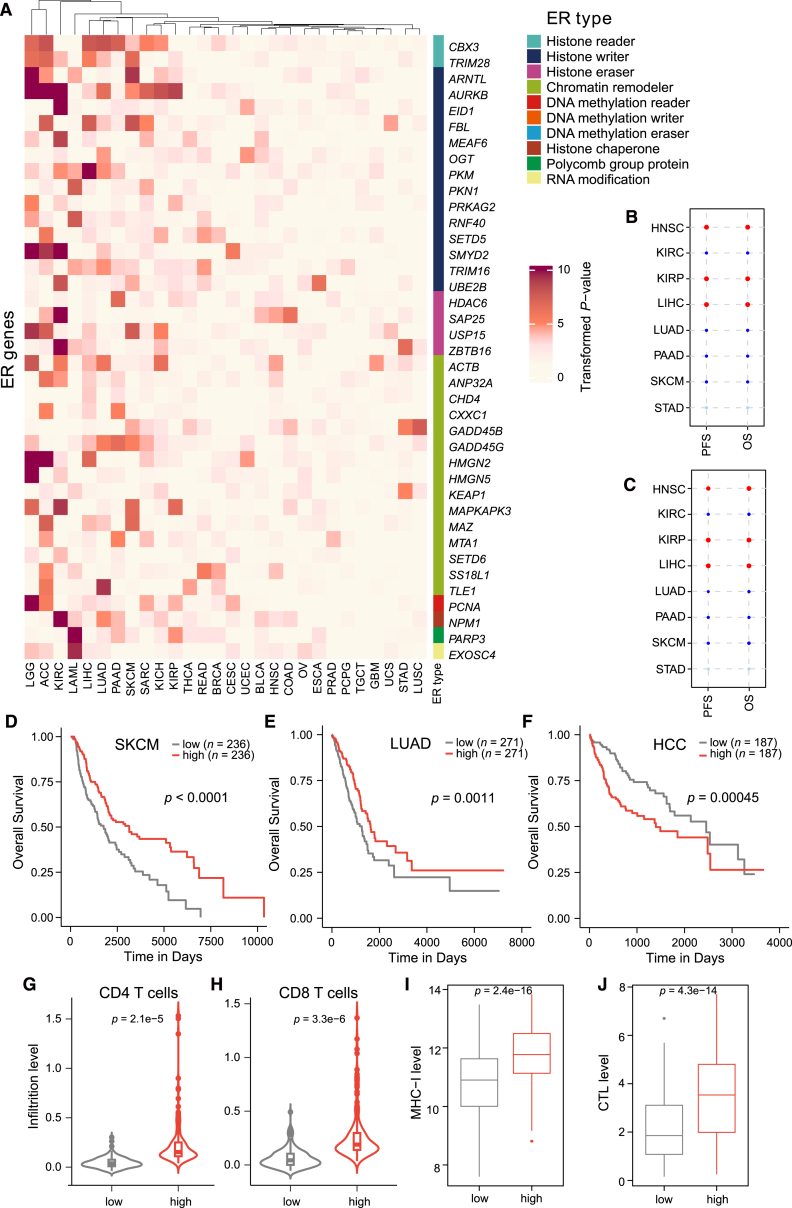
The ER signature scores can predict cancer prognosis in different cancer types (A) Heatmap of transformed survival *p* values of the DEGs identified only by dysregulation score in 28 cancer types. Survival *p* values are computed based on the prognosis of tumor patients that are associated with high (top 50%) vs. low (bottom 50%) ER levels. Heatmaps are plotted based on a log_10_-transformation with a pseudocount. (B) Univariable hazard ratios (HRs) of the ER signature for overall survival (OS) and progression-free survival (PFS). (C) Multivariable HRs of the ER signature for OS and PFS while adjusting for age, gender, and tumor stage. Dot size is proportional to HR. The red dot represents HR > 1 and *p* < 0.05, the blue dot represents HR < 1 and *p* < 0.05, and the light blue represents HR < 1 and *p* > 0.05. (D) Kaplan-Meier curves of the ER signature and OS in SKCM. (E) Kaplan-Meier curves of the ER signature and OS in LUAD. (F) Kaplan-Meier curves of the ER signature and OS in HCC. (G) Violin plots showing the distribution of infiltration levels of CD4 T cells in two groups with different ER signature scores for SKCM. (H) Violin plots showing the distribution of infiltration levels of CD8 T cells in two groups with different ER signature scores for SKCM. (I) The boxplot showing the MHC-I expression in two groups with different ER signature scores for SKCM. (J) The boxplot shows the cytotoxic T-lymphocyte (CTL) expression in two groups with different ER signature scores for SKCM. Two-sided Wilcoxon’s rank-sum test determines significance between two groups. HCC, hepatocellular carcinoma; LUAD, lung adenocarcinoma; SKCM, skin cutaneous melanoma. Depicted *p* values in (D)–(F) are from log rank tests.

We were interested in whether the expression features of the DEGs can predict cancer prognosis. We identified 222 DEG-cancer associations (log rank test, *p* < 0.05) that were significantly related to cancer prognosis from 1,081 cancer-specific DEGs (Figure 7A). Different ER types showed minimal differences in cancer prognosis (Figure 7A).

We further constructed a gene signature based on the DEGs with significant prognosis association for each cancer type (Figure S6). With the median value of signature scores as the cutoff, the cancer-specific patients were grouped into high- and low-score subgroups. The univariate and multivariate Cox regression analysis showed that the ER signatures can predict patient overall survival (OS), controlling for age, gender, and tumor stage (log rank test, *p* < 0.05, Figures 7B and 7C). Consistently, the signatures can also predict progression-free survival (PFS), comparable to OS (Figures 7B and 7C). Interestingly, we found significant associations between the ER signature and cancer patient outcomes in specific cancer types (Figures 7D–7F). For example, in hepatocellular carcinoma (HCC), the cancer patients with higher signature scores were associated with significantly shorter OS compared with those with lower signature scores (Figure 7F), whereas in skin cutaneous melanoma (SKCM) and lung adenocarcinoma (LUAD), a higher signature score indicated a better OS than a lower signature score (Figures 7D and 7E).

In terms of cancer immune-related features, the ER signature score was positively correlated with and infiltration levels of anti-tumor immune-associated cells in SKCM, such as CD4 T cells and CD8 T cells (Figures 7G and 7H). Higher cytotoxic T-lymphocyte (CTL) and major histocompatibility complex class I (MHC-I) expression levels were observed in the higher-score group (*p* < 0.001, Figures 7I and 7J).

Furthermore, we investigated the association of risk groups with somatic mutations for cancer-specific driver genes and found several significant associations (Figure S7). For SKCM patients, *BRAF* had a higher mutational fraction in the higher-score individuals, while *TP53* and *NF1* had a higher mutational fraction in the lower-score individuals (Figure S7A). For the lung adenocarcinoma (LUAD) patients, the mutational rate in *DACH1* was higher in the higher-score individuals, while *CDKN2A* and *SMARCA4* were higher in the lower-score individuals (Figure S7B).

### DEG-based gene signatures can improve the prediction performance of the efficiency of cancer immunotherapy

**Figure 8:**
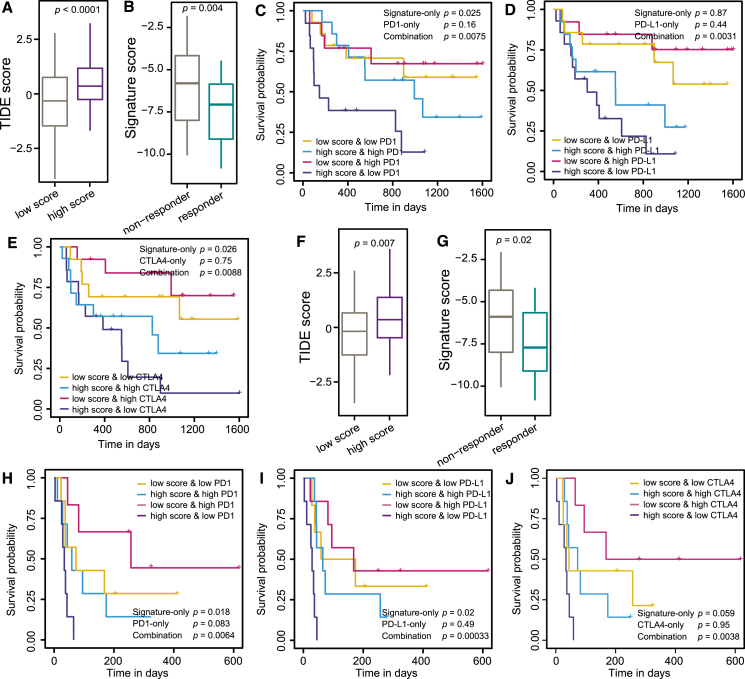
The ER signature scores can better predict the efficiency of ICB treatment in cancer patients (A) Boxplots showing differences of TIDE scores for the melanoma SKCM patients with high- and low-signature scores. (B) Differences in the risk signature scores between responders and non-responders among the SKCM patients receiving ICB treatment. Kaplan-Meier analysis of progression-free survival for the SKCM patients stratified by high- and low-risk signature scores and (C) PD1 expression levels, (D) PD-L1 expression levels, and (E) CTLA4 (cytotoxic T-lymphocyte-associated protein 4) expression levels. (F) Boxplots showing differences in the TIDE scores between high- and low-risk lung adenocarcinoma (LUAD) patients. (G) Differences in risk signature scores between responders and non-responders among the LUAD patients receiving ICB treatment. Kaplan-Meier analysis of progression-free survival for the LUAD patients stratified by high- and low-risk signature scores and (H) PD1 expression levels, (I) PD-L1 expression levels, and (J) CTLA4 expression levels. Two-sided Wilcoxon’s rank-sum test determines significance between two groups. Depicted *p* values in (C)–(E) and (H)–(J) are calculated by log rank tests.

We obtained transcriptomic data from SKCM and LUAD cancer cohorts receiving immune checkpoint blockade (ICB) immunotherapy from two previous studies to independently evaluate the clinical validity of the SKCM and LUAD ER signatures.^38^^,^^39^ Given the significant associations between different groups and patient survival/immune activity, we next investigated whether the ER signature could help better predict the efficiency of cancer immunotherapy. When applying the ER signature to the SKCM or LUAD patients receiving anti-PD1 therapy, higher-score patients showed higher TIDE scores (Figures 8A and 8F).^20^^,^^40^ We also observed higher signature scores in the non-responders than the responders (two-sided Wilcoxon’s rank-sum test) after anti-PD1 treatment, demonstrating that higher-score patients are less likely to benefit from ICB treatment (Figures 8B and 8G).

Elevated expression levels of immune checkpoints, such as PD-1, programmed cell death ligand 1 (PD-L1), and cytotoxic T-lymphocyte-associated protein 4 (CTLA-4), have been associated with enhanced clinical responses to ICB therapy in cancer patients, as demonstrated in prior studies.^41^^,^^42^ We previously indicated that cancer patients with higher signature scores showed lower expression of checkpoints (e.g., PD1, PD-L1, and CTLA4).^26^ We therefore stratified the SKCM or LUAD patients into four different combinations of risk signature and checkpoint expression group. We observed that dichotomous signature scores can efficiently separate SKCM patients with survival differences, which is more efficient than using dichotomous checkpoint expression levels or mutational burden only (Figures 8C–8E). Specifically, the DEG-based signature score can predict the OS time of SKCM patients better than PD-1 expression levels (Figure 8C, log rank test *p* = 0.025 for significant score and *p* = 0.16 for PD-1 expression). The combination of signature score and PD-1 expression can even increase the prediction performance (*p* = 0.0075), suggesting a synergistic effect of the two scores. We also observed similar effect of combining signature score and PD-L1 expression (Figure 8D, log rank test *p* = 0.44, 0.87 and 0.0031 for signature score, PD-L1 expression, and the combination, respectively), and combining signature score and CTLA4 expression (Figure 8E, log rank test *p* = 0.026, 0.75, and 0.0088 for signature score, CTLA4 expression, and the combination, respectively). We also observed that dichotomous signature scores can efficiently separate LUAD patients with survival differences, which is more efficient than using dichotomous checkpoint expression levels or mutational burden only (Figures 8H–8J).

### Identification of putative medicines that can perturb the expression of DEGs

The efficient selection of potential compounds/medicines and their derivatives for gene targets may be important in cancer therapy. A plethora of research has indicated that medicines or compounds have the potential to reverse target gene expression in cancer. For example, iron chelators have been reported to attenuate, promote cell-cycle arrest, and activate TSGs.^43^ Several *TOP2* inhibitors were approved by the US Food and Drug Administration, such as etoposide and doxorubicin.^44^ In addition, the inhibition of KDM4 by pan-KDM4 inhibitor QC6352 can also repress cancer cell line proliferation.^45^ Existing drugs developed for diseases other than cancer can also be repurposed for cancer treatment, for example, linagliptin.^46^^,^^47^ Linagliptin can physically interact with the genes *CHRM1*, *FAP*, *ALDH1A1*, and *PDE6D* to modulate their gene expression patterns.^47^ However, few compounds/medicines targeting ERs and modulating their expression patterns had been identified until recently. It was therefore interesting to identify compounds or medicines that could reverse the expression of differentially expressed ER genes. To this end, we identified the statistically significant relationships between our DEGs and compounds/medicines based on the CRowd Extracted Expression of Differential Signatures (CREEDS) dataset. As a result, we found 58 compounds/medicines are significantly associated with 77 DEGs based on the cutoff of *q*-value < 0.05 and absolute log_2_FC > 1 in the CREEDS dataset (Figure S8). Specifically, 25 compounds/medicines were approved as anticancer and chemotherapy drugs, 20 compounds/medicines were evaluated solely through molecular biology experiments without further clinical trials, and 13 compounds/medicines lacked available literature evidence (Figure S8). The detailed information of compounds/medicines that increased the expression of downregulated ER genes or decreased the expression of upregulated ERs was available in Table S4, from which we found doxorubicin was the highest-ranking medicine that can reverse the expression of upregulated or downregulated ER genes (according to the largest 10 absolute FC), which was consistent with a previous study.^37^ Altogether, we identified candidate compounds/medicines with the potential for repurposing in future cancer therapies targeting ERs.

## Discussion

Previous studies have elucidated the epigenetic regulatory functions of specific ER genes in cancer over the past 3 decades.^48^^,^^49^ Nevertheless, a thorough understanding of the dysregulation of ER genes in oncogenesis remains incomplete. In this study, we identified 272 cancer-specific DEGs from a cohort of 690 human ER genes by analyzing the transcriptomic data across 28 human cancer types. Our functional genomic analyses indicate that the genetic disruption of epigenetic domains may constitute one of the primary mechanisms underlying ER gene dysregulation in cancer (Figure 5). However, further investigation is required in future studies to substantiate this hypothesis. The signature scores derived from the dysregulation of ER genes serve as a prognostic indicator for cancer patients as well as patients receiving immunotherapy (Figure 8). In particular, the prognostic outcomes of cancer patients are correlated with signature scores derived from these DEGs (Figure 8).

A key methodological advance of this study is the introduction of the DS score, a simple method tailored explicitly for differential expression calling in large, population-level cancer cohorts.^50^ Existing algorithms such as DESeq2 and edgeR assume negative-binomial distributions that are frequently violated by the heavy-tailed, highly skewed count distributions observed when hundreds of tumor samples are analyzed. These violations inflate the false discovery rate and, in our data, limma and edgeR yielded ∼10-fold more candidate genes than DESeq2 (FDR = 5%) and the DS metric. Although a comprehensive benchmarking study is beyond the scope of the present work, the genes uniquely identified by the DS score showed a significantly higher enrichment for the known cancer drivers cataloged in the CGC and IntOGen databases (Figures 3C and 3F). Functional experiments preliminarily validate the cancer-driving potential of four DS-score-specific candidates (Figure 4).

Our analyses reveal that the expression levels of hundreds of ERs were modified in human tumor samples, as indicated by the DS score (Figure 1). This alteration encompasses both cancer-type-specific and pan-cancer recurrent dysregulation. Notably, *TOP2A* and *ZBTB16* are the most frequently upregulated and downregulated ER genes, respectively, across various cancer types. However, they have been extensively studied in only a limited number of these cancers. Future research can also elucidate the roles of other less-characterized DEGs within specific cancer types and explore their potential as therapeutic and medicine targets.

The identification and characterization of loss-of-functional mutations are crucial for developing targeted therapies that can improve patient outcomes. The role of loss-of-functional mutations in specific ER genes has been shown to contribute to cancer progression.^51^ This underscores the importance of focusing on not only well-known cancer driver genes but also on ER genes that may be frequently mutated in epigenetic domains. The role of loss-of-functional mutations (e.g., nonsense and missense mutations) in specific ER genes has been shown to contribute to cancer development and progression, making them attractive targets for therapeutic intervention. For instance, gain-of-functional mutations in the gene encoding the enhancer of zeste homolog 2 have been shown to lead to a genome-wide increase in histone-3 Lys27 trimethylation, thereby inactivating multiple TSGs. Pharmacological inhibition of mutated *EZH2* has been demonstrated to restore gene expression and chromatin interactions, highlighting its potential as a therapeutic target.^52^ In addition, inhibitors targeting the histone methyltransferase DOT1L and the demethylase LSD1 are being explored for their therapeutic potential, as they can modify the epigenetic landscape of cancer cells and influence gene expression without disrupting the DNA sequence.^53^ Our findings indicate that epigenetic domains within DEGs may present a higher somatic mutation burden compared with non-epigenetic domains (Figure 5). Although preliminary, these results underscore the potential significance of epigenetic domains in the development of cancer therapies and clinical interventions. Continued research into the mechanisms underlying loss-of-function mutations in ER genes is essential for advancing personalized and more effective cancer treatment strategies.

Our study reveals that a significant proportion of DEGs exhibits limited connectivity within the human PPI network, an observation that is contrary to our expectation (Figure 6). This finding implies that ER expression perturbation by pharmacological interventions might pose a reduced risk to cellular viability compared with targeting other genes. A plausible explanation for this phenomenon is that ERs that may interact with the same domain substrate are generally tissue-specific to fulfill specific developmental regulation (Figure S3).^3^ Therefore, the dysregulation of a specific tissue-specific ER may not necessarily be harmful to the cells. In contrast, the PPI network hub genes appear to play a more pivotal role in the assembly of the gene modules (Figure 6A). In addition, the modules formed by ER genes and hub genes are enriched for biological functions associated with cancer and immune responses, suggesting that dysregulated ER genes may exert their effects on downstream cancer-related pathways, especially cancer immune regulation, through interactions with hub genes.

ERs, such as the DNMT inhibitors and HDAC inhibitors, have been widely used in epigenetic therapy, enhancing the immunogenicity of tumors and improving the efficacy of cancer treatment. However, the clinical application of these agents is complicated by the issues related to their efficacy and potential toxicities. One of the primary challenges is the limited efficacy of ER inhibitors when used as monotherapies. Studies have shown that while these agents can alter the tumor microenvironment and enhance immune responses, their effects are often insufficient to produce significant clinical outcomes on their own. The inefficiency of monotherapies has led to the exploration of combination therapies, where ERs can be used alongside ICIs or other forms of immunotherapy to achieve a synergistic effect.

Combination strategies have long served as a foundational principle in oncology, aiming to enhance therapeutic efficacy by leveraging synergistic effects between targeted medicines and chemical agents. The use of ERs in conjunction with tumor immunotherapy represents a promising approach within the realm of combination cancer therapies.^25^ In recent years, the integration of ERs with tumor immunotherapy has yielded significant advancements in cancer treatment, leading to improved therapeutic outcomes.^54^ These include enhanced disease remission rates, prolonged disease-free and overall survival, and a modest reduction in treatment-related adverse events. For instance, combining ERs with checkpoint blockade antibodies has shown promise in the preclinical models, suggesting that such combinations could potentiate anti-tumor immune responses and improve clinical outcomes.^55^ However, the clinical effectiveness of such regimens can vary markedly, depending on the tumor type, molecular profile, and the specific therapeutic agents employed. Tailoring combination therapies to tumor-specific contexts remains essential to optimizing patient outcomes and minimizing adverse effects. In this work, we have identified ER gene signatures with potential clinical applicability (Figures 7 and 8). Furthermore, we have indicated that ERs coupled with immune checkpoints showed an even greater performance than those used alone (Figure 8). Mechanistically, epigenetic therapies enhance tumor immunogenicity and modulate the immunosuppressive tumor microenvironment through multiple immune-related pathways, especially the antigen processing and presentation pathway. These include the upregulation of major histocompatibility complex molecules, co-stimulatory signals, and tumor-associated antigens. Additionally, epigenetic modulation may promote cytokine secretion and alter immune signaling by increasing the expression of immune checkpoint molecules such as CTLA-4, PD-1, and PD-L1 (Figure S9). This favorable regulation of immune checkpoint expression suggests that epigenetic perturbation can both stimulate and regulate anti-tumor immunity, underscoring the need for further investigation into their precise mechanisms of action in the tumor immune landscape. Altogether, ERs are pivotal in modulating immune responses and mediating anti-tumor effects, particularly by overcoming the mechanisms of immune evasion within the context of immunotherapy.

While the combination of ERs and tumor immunotherapy holds significant therapeutic promise, the clinical application of such combination therapies remains constrained by specific limitations and patient-dependent variability. First, only a subset of patients derives meaningful benefit from these strategies. For instance, anticancer agents such as azacytidine and bevacizumab, which target ERs, may fail to elicit the expected anti-tumor effects in patients with solid tumors who have previously received immunotherapy.^56^ While combination approaches are feasible and show promise in certain malignancies, they do not universally enhance therapeutic efficacy and, in some cases, may exacerbate treatment-related toxicity. In high-risk myelodysplastic syndrome, for example, the combination of azacitidine with the ICI durvalumab resulted in greater toxicity without any corresponding improvement in clinical outcomes compared with azacitidine monotherapy.^57^ Addressing these limitations will require a deeper understanding of the epigenetic regulatory mechanisms governing tumor immunity, which may inform the development of safer and more effective clinical strategies. Notably, the DEGs identified in our study may serve as novel epigenetic targets, offering a means to circumvent the severe toxicity and off-target effects associated with earlier generations of epigenetic inhibitors, such as DNMT and HDAC inhibitors. By elucidating the roles of these DEGs, our study contributes to a deeper understanding of the molecular mechanisms underpinning cancer progression. Moreover, there is growing interest in exploring synergistic strategies that combine epigenetic therapies with advanced immunotherapeutic modalities such as chimeric antigen receptor T cell therapy, adoptive cell transfer, and tumor vaccines. A limitation in this study is that we only conducted a preliminary validation of four candidate epi-driver genes in cancer cell lines, and the identified DEGs and related signatures require further validation through *in vivo* studies.

While our findings underscore the therapeutic promise of combining tumor immunotherapy with epigenetic modulation, a significant challenge in implementing ER-based precision oncology lies in the precise and safe delivery of therapeutic agents at nanoscale concentrations to tumor sites. Future research should focus on optimizing delivery systems to enhance the bioavailability and targeting efficiency of these compounds, thereby improving clinical outcomes. Future research may also focus on the development of next-generation epigenetic agents that selectively target immune evasion pathways, to improve therapeutic precision and reduce off-target effects. Overall, the integration of tumor immunotherapy with targeted epigenetic interventions represents a promising avenue in cancer therapy, with the potential to reshape future treatment paradigms substantially.

In our study, we also identified dozens of candidate compounds capable of reversing the expression of DEGs in cancer cell lines. These agents, many of which are existing pharmaceuticals, hold potential for repurposing in clinical oncology. Our findings show that a substantial proportion of DEGs, including both oncogenes and tumor suppressors, can be modulated by specific compounds (Figure S8). Doxorubicin, a widely used chemotherapeutic agent known to regulate DNA damage, cell-cycle arrest, and apoptosis, can perturb the expression of several cancer driver genes (*GADD45B*, *UHRF1*, *CDC6*, etc.) that are involved in cell-cycle regulation, from this high-throughput data analysis. Consistent with that doxorubicin prevents ubiquitin-like with PHD and ring finger domains 1 (UHRF1) from binding to hemi-methylated DNA, our data indicate that doxorubicin-perturbed cancer-related genes, particularly those involved in cell-cycle regulation, represent a possible aspect of its mechanism of action.^58^ It is important to note that most of these compounds in Figure S8 have been validated only in preclinical settings, and their safety and efficacy must be confirmed through rigorous *in vivo* studies. Ongoing clinical trials aim to optimize dosing strategies and identify predictive biomarkers that can enhance patient responsiveness while minimizing toxicity. Although the present work does not address whether the investigated compounds could modulate immune checkpoint expression, a previous study indicated that doxorubicin, a well-established chemotherapeutic agent, has also been observed to transcriptionally upregulate PD-L1, which is inversely correlated with the expression of miR-140, a microRNA that targets PD-L1 mRNA.^59^ In addition, decitabine, an epigenetic modifier, has been shown to enhance PD-L1 expression through DNA hypomethylation, thereby improving the efficacy of PD-L1 blockade in colorectal cancer models.^60^ Therefore, the combination of chemotherapeutic agents with immune checkpoint inhibitors might be a promising strategy to enhance anti-tumor immunity. By understanding the mechanisms through which these agents influence immune checkpoint expression, researchers can design more effective combination therapies that harness the full potential of both chemotherapy and immunotherapy. Such a strategy could lead to improved outcomes for patients with various types of cancer, particularly those who are resistant to conventional treatments.

In summary, we have identified and characterized 272 cancer-specific DEGs in publicly available transcriptomic data from a large number of pan-cancer human tumor samples based on a proposed dysregulation score, of which four novel DEGs are validated by cell-line-based functional assays, including CCK-8 and transwell assays as a proof-of-concept, enhancing the understanding of the role of ER gene dysregulation in cancer. The knockdown of DEGs can alter the mRNA expression levels of immune checkpoint genes such as PD-L1 and CTLA4, suggesting their immune regulation potential. We demonstrate that DEG-based signature score in conjunction with immune checkpoints can predict the efficiency of cancer immunotherapy better than using immune checkpoints alone, highlighting the potential of DEGs as diagnostic targets. These identified DEGs represent a valuable resource for evaluating cancer prognosis and immunotherapy, as well as for the development of ER-targeted therapeutics.

## Materials and methods

### Datasets

Ethical approval was not required for this study, as all omics data were obtained from publicly available databases. We downloaded the transcripts per kilobase million (TPM) expression data in humans from the UCSC (University of California, Santa Cruz) XENA Project website, which includes the gene expression data for TCGA, TARGET, and GTEX cohorts, including 19,131 human samples (18,393 tumor and 738 normal samples).^61^ From the same website, we downloaded the MuSE masked somatic mutation data and the survival information of 10,061 TCGA cohorts from the XENA Project website.^62^ Perturbed gene expression data, derived from thousands of RNA-seq datasets encompassing 1,458 genes, were obtained from the GPSAdb database.^63^ The perturbed gene expression data derived from thousands of RNA-seq datasets allowed us to explore the influence of ER gene perturbation upon expression of the core genes in the antigen processing and presentation pathway. We obtained the list of 690 human ER genes with ER subtype annotations from a previous study, where genes with annotated “histone_type” or “methylator_type” were used in this work.^18^

### Analysis of ER expression and differential expression in pan-cancer samples

We performed the gene differential expression analysis for tumor-normal samples based on the processed gene expression data from XENA.^32^^,^^61^ The expression data were log_2_-transformed (with a pseudocount). For each cancer type and ER gene, we then calculated the log_2_FC for median log_2_ expression levels in tumor and normal patient samples. For a specific cancer type, we extracted residuals from the *rlm* function results using the MASS R package (version 7.3) for the gene expression of paired tumor and normal samples, and termed it DS, as was proposed by a previous study.^64^ DS score is a more conservative dysregulation score than the FC ratio. The DEGs were defined by ≥ 2.5 standard deviations of |DS|, corresponding to the outer 2.1% of a standard normal distribution. Genes exceeding this threshold were considered significantly dysregulated, with positive dysregulation scores indicating upregulation and negative scores indicating downregulation. For visualization, DS values were centered and scaled prior to heatmap construction using the ComplexHeatmap R package (version 2.16.0).^65^

### Cell culture and transfection

The human melanoma cell lines SK-MEL-2 (catalog no. CL-0706) and A549 (catalog no. CL-0016) were purchased from Procell Life Science & Technology Co., Ltd. (Wuhan, China). Cells were cultured in Dulbecco’s modified Eagle’s medium (DMEM) and maintained at 37°C in a humidified incubator with 5% CO_2_. For transfection, cells were seeded at a density of 1 × 10^6^ cells per well in six-well plates and incubated for 24 h.

Single-stranded siRNAs targeting the indicated human genes, along with NC siRNA duplexes, were synthesized by RiboBio Co., Ltd. (Guangzhou, China) using standard phosphoramidite chemistry. A total of 100 nM of siRNAs were transiently transfected into cells with Lipo8000 (catalog no. C0533, Beyotime, China). pcDNA3.1(+) vectors (Invitrogen, Carlsbad, CA, USA) carrying candidate genes were synthesized by GenScript (Nanjing, China) and co-transfected with the corresponding siRNAs to assess whether exogenous expression of the encoded proteins can rescue the observed cellular phenotypes caused by siRNAs. The transfection medium was replaced with complete growth medium 6–8 h post-transfection. At 48 h after transfection, cells were harvested for subsequent experimental assays.

### Quantitative real-time PCR

Long total RNAs (>200 nt) were extracted using the RNAeasy Animal RNA Isolation kit (#R0032, Beyotime, China). The concentration of RNA was determined using a DS-11FX spectrophotometer (DeNovix, Wilmington, DE, USA), then 1 μg total RNA for each sample was reverse transcribed to complementary DNA (cDNA) using the HiScript IV RT SuperMix for qPCR (+gDNA wiper) kit (#R423-01, Yeasen, China). The reactions were incubated for 15 min at 37°C, followed by 5 s at 85°C. After cDNA synthesis, cDNA amplifications were carried out on a LightCycler 480 II instrument (Roche, Basel, Switzerland) based on a Taq Pro Universal SYBR qPCR Master Mix kit (#Q712-02, Vazyme, Nanjing, China) according to the provided manual. The primers were synthesized by RiboBio (RiboBio Co. Ltd., Guangzhou, China) and are summarized in Table S5. The reactions were as follows: 95°C for 30 s (hold stage), followed by 40 cycles of 95°C for 10 s, 60°C for 30 s (PCR stage), then 95°C for 15 s, 60°C for 15 s, 95°C for 15 s (melt-curve stage). The mRNA expression of each investigated gene was normalized to that of *GAPDH*. Gene expression levels were quantified as threshold cycle values, while relative quantification levels of genes were normalized with *GAPDH* using the 2^−ΔΔ*C*t^ method.

### CCK-8 assay

Cell proliferation of SK-MEL-2 and A549 cells was assessed using the CCK-8 (#GK10001, GLPBIO, China). Cells were seeded in 96-well plates at a density of 3,000 cells per well in 100 μL of DMEM. After incubation at 37°C in a 5% CO_2_ humidified atmosphere for 24 h to allow cells to reach the logarithmic growth phase, CCK-8 assays were conducted at 0, 24, 48, and 72 h post-seeding. At each time cycle, 10 μL of CCK-8 reagent was added to each well, followed by incubation for 3 h in the original culture medium. Cell viability was then quantified by measuring the absorbance at 450 nm using a microplate reader (Epoch2, BioTek, Beijing, China). Optical density values were recorded and used to evaluate relative cell proliferation.

### Transwell invasion assay

The Matrigel matrix glue (#356234, Corning Incorporated, USA) was placed in the refrigerator at 4°C until completely melted. The standard Matrigel glue was diluted on ice to 200 μg/mL with serum-free medium, and then 100 μL of the diluted Matrigel was placed in 24-well transwell plates (#3422, Thermo Fisher Scientific, USA). The plates were then incubated at 37°C (5% CO_2_) for 3 h until the Matrigel clotted. The transfected cells were trypsinized and washed twice with PBS, then resuspended in serum-free medium. Cell suspension (200 μL) was seeded in the upper chamber of the transwell, and 800 μL of complete medium was added to the lower chamber. After incubating for 48 h, the cells in the upper chamber were washed with PBS twice and fixed with 4% paraformaldehyde (#BL539A, Biosharp, Hefei, China) for 30 min, the paraformaldehyde was then removed and the cells that did not pass through the membrane were wiped with cotton swabs, following by staining with 0.1% crystal violet (Sigma-Aldrich, St. Louis, MO, USA) for 30 min. The migratory cells adhering to the lower surface of the membrane were photographed. Each group was randomly selected to take pictures with a microscope and cells were counted by ImageJ software.

### Survival analyses

We performed survival analyses based on the TCGA survival data. We divided all the cancer patients into a high-expression group (higher compared with average expression) and a low-expression group (lower compared with average expression) based on gene expression for a particular ER gene. We also matched the individuals with survival information. We used the survminer R package (version 0.4.9) to generate survival curves for each gene and cancer type and obtained the survival *p* values. Heatmaps of the survival *p* values were generated based on the log transformation of *p* values (with a pseudocount), that is, log(1/*p* value+1) by the ComplexHeatmap R package (version 2.16.0).^65^

### Gene set overrepresentation and enrichment analyses

Gene set overrepresentation analyses for the dysregulated genes were performed by the Enrichr website.^66^ In addition, the public data that were used to quantify the enrichment of DEGs in the duplicated genes and the dysregulated genes were shown as follows: (1) a cancer driver gene set including 1,172 DORGE predicted cancer driver genes^37^; (2) the PPI data downloaded from BioGRID v4.4.209 (https://thebiogrid.org/).^67^ The homodimer and duplicated interactions in the BioGRID PPI network were removed, resulting in a total of 724,360 non-redundant interactions; (3) 3,804 HKG genes from https://www.tau.ac.il/∼elieis/HKG/; (4) essential genes (more than two entries) and the duplicated genes that were also obtained from the OGEE database.^68^ Gene enrichment analyses were done by one-sided Fisher’s exact test (fisher.test function of stats package version 4.3.1 in R). Functional terms with the *p* values adjusted by false discovery rate <0.05 were considered significant.

### Somatic mutation analyses

We analyzed somatic mutations in DEGs to investigate their mutational distribution pattern. The somatic mutations were also downloaded from the UCSC XENA Project website.^61^ We used the G3viz R package (version 1.2.0) to generate lolliplots.^69^

### PPI network analyses

We performed the PPI module analysis using the Metascape website for the subnetwork including DEGs in cancer and PPI-hub genes.^70^ The MCODE algorithm was applied to identify the closely connected modules from the DEG and hub subnetwork.^71^ We defined the hub genes as genes with degree higher than top 1% degrees in the entire BioGRID PPI network. We obtained 176 hub genes while excluding 22 DEGs. Network metrics like degree, betweenness centrality, and closeness centrality were calculated by the igraph package (version 1.3.1) in R.

### Generation of the ER signature

To quantify ER dysregulation patterns in individual cancer types and investigate the clinical relevance of the DEGs, we established a DEG-based signature in each cancer type. By multivariate Cox proportional hazards model analysis (survival R package, version 3.5.0), DEGs significantly correlated with patients' overall survival (OS), adjusting for age, gender, and tumor stage, were retained (*p* < 0.05). For a specific patient, we obtained an ER signature score according to the risk-weighted sum expression: $signature\;score=\sum_{i=1}^{n}coef\left({ER}_{i}\right)*\exp\left({ER}_{i}\right)$, where *coef*(*ER*_*i*_) indicates the Cox coefficients for the *i*th ER and *exp*(*ER*_*i*_) indicates the expression levels for the *i*th ER. In a specific cancer type, higher- and lower-score patient subgroups were defined based on the median value of signature scores for the patients.

### Cancer immune analysis

We obtained the estimated abundance of tumor-infiltrating immune cells from TIMER, for example, CD4 T and CD8 T cells.^72^ The average expression of HLA-A/B/C and B2M was used to represent the MHC-I expression. The CTL level was calculated from the average gene expression of CD8A, CD8B, GZMA, GZMB, and PRF1. The tumor immune dysfunction and exclusion (TIDE) method was used to estimate the potential response of patients to ICB therapy, based on the gene expression profiles specific to each cancer type.^20^

### Drug response analysis

Drug response data from the CREEDS collections were retrieved from the Drug Gene Budger database.^73^ Only statistically significant drug-gene interactions were retained, based on a threshold of *q*-value < 0.05 and fold change >2 (or <1/2). DEGs that were associated with at least three compounds, as well as compounds that are associated with at least four DEGs, were kept for further analysis.

### Statistical analyses

Statistical analysis of bar or line plots for molecular experiments was carried out using R computing system. Results are represented as the mean ± standard error of the mean. An unpaired *t* test was performed to analyze the difference between the two experimental groups. Statistical significance in boxplots was determined by a two-sided Wilcoxon rank-sum test.

## Data and code availability

The datasets generated and/or analyzed during the current study are available in the UCSC XENA Project (https://xena.ucsc.edu/) and the Gene Expression Omnibus (GEO; http://www.ncbi.nlm.nih.gov/geo/) unless otherwise specified. The processed datasets supporting the conclusions of this article are included within the supplemental files. The supplemental data supporting the findings of this study are available from the corresponding author upon reasonable request. The omics data processing codes are available at https://figshare.com/projects/Prediction_of_dysregulated_epigenetic_regulator_genes/253955.

## Acknowledgments

J.L. is grateful for the encouragement and support provided by his wife, Chengzhi Qu. This work was supported by the Wenzhou Institute, 10.13039/501100011332University of Chinese Academy of Sciences’ startup fund under grant number WIUCASQD2021006 and the Zhejiang Key Laboratory of Soft Matter Biomedical Materials (number: 2025ZY01036, 2025E10072).

## Author contributions

J.L. participated in data curation, formal analysis, funding acquisition, project administration, supervision, writing – original draft, and writing – review and editing; H.Z. participated in formal analysis; J.Z. contributed to conceptualization, formal analysis, investigation, methodology, and writing – review and editing; Z.F. contributed to conceptualization and writing – review and editing.

## Declaration of interests

The authors declare that they have no competing interests.

## Supplemental information

Document S2. Tables S1–S5

Document S3. Article plus supplemental information
