# Supplementary material for: The dysregulation score method identifies epigenetic regulator genes that predict cancer prognosis and efficiency of cancer immunotherapy
Source: Mol Ther Nucleic Acids. 2025 Nov 19;36(4):102781. doi: 10.1016/j.omtn.2025.102781 (PMC12718196; doi:10.1016/j.omtn.2025.102781)
Supplement: Document S1. Figures S1–S9 [file mmc1.pdf]

OMTN, Volume *36*

Supplemental information

The dysregulation score method identifies epigenetic regulator genes that predict cancer prognosis and efficiency of cancer immunotherapy

Jie Lyu (吕杰), Hao Zhang (张昊), Jinjin Zhong (钟金金), and Zhen Feng (冯振)

Supplemental information

Supplemental Figures

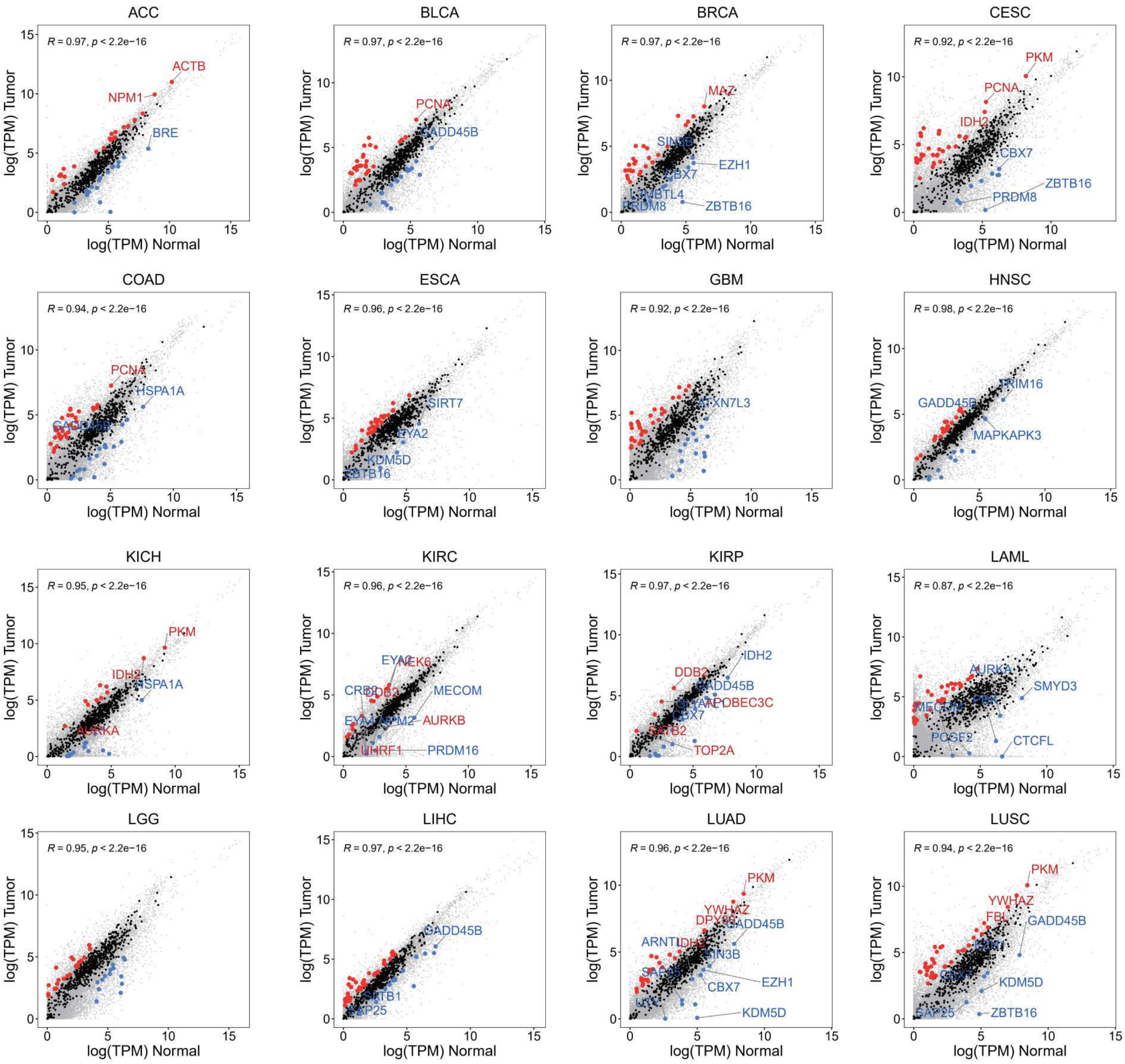

Figure S1. Scatterplots of the $\log_2$TPM values for ER genes in the human tumor and matched normal samples in 16 cancer types. ER genes are highlighted in black, of which dysregulated ER genes are further highlighted, that is, upregulated in red or downregulated in blue. Non-ER genes are shown in gray color. Pearson correlation coefficient R is used to evaluate the correlation between paired samples (cor function in R) for each cancer type, and P-value is calculated by Test for Association/Correlation Between Paired Samples (cor.test function in R). TPM, transcripts per kilobase million.

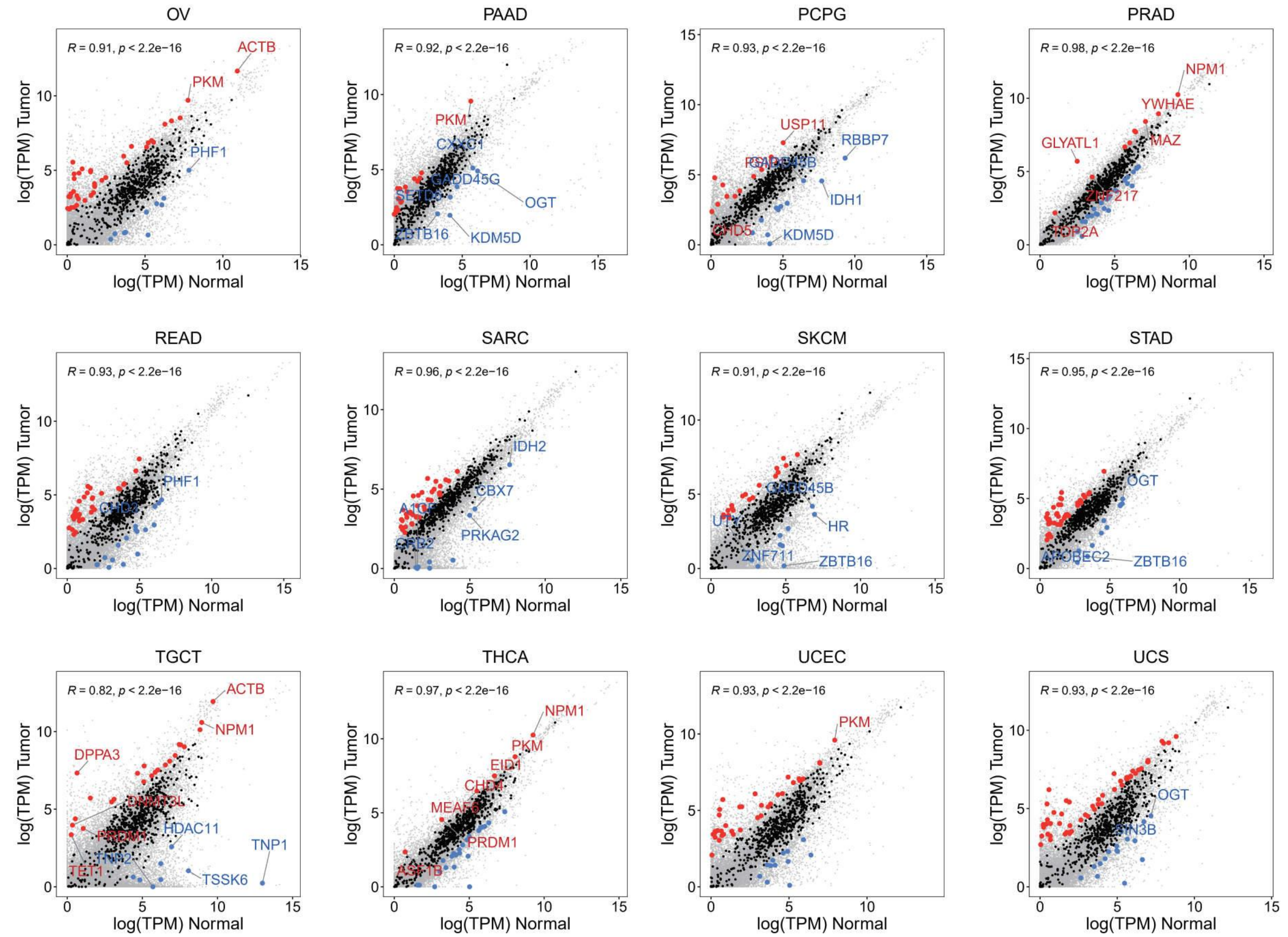

Figure S2. Scatterplots of the log_2TPM values for ER genes in the human tumor and matched normal samples in 12 cancer types. ER genes are highlighted in black, of which dysregulated ER genes are further highlighted, that is, upregulated in red or downregulated in blue. Non-ER genes are shown in gray color. Pearson correlation coefficient *R* is used to evaluate the correlation between paired samples (cor function in R) for each cancer type, and *P*-value is calculated by Test for Association/Correlation Between Paired Samples (cor.test function in R). TPM, transcripts per kilobase million.

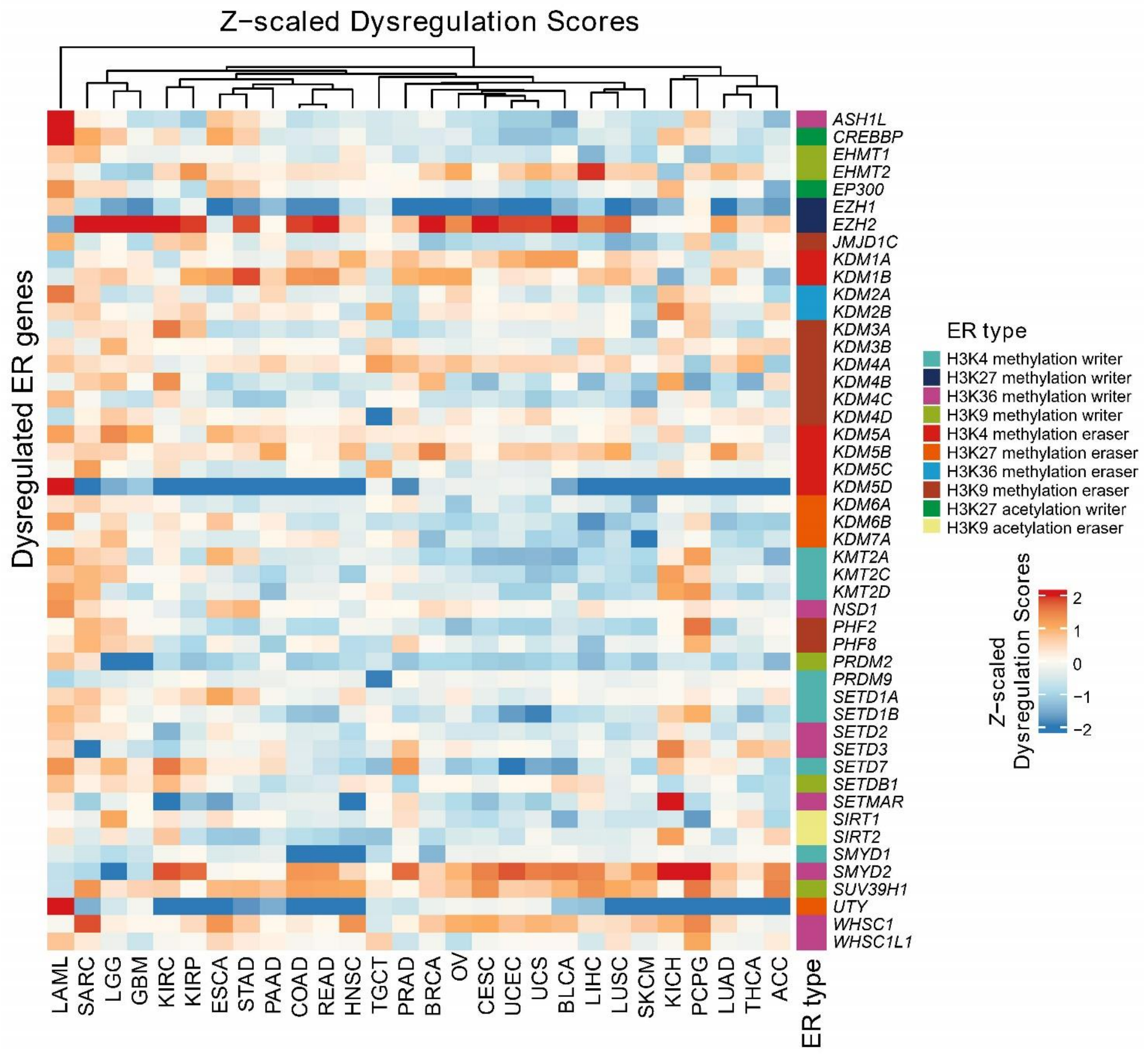

Figure S3. Heatmap of dysregulation scores of dysregulated ER genes (DEGs) with different epigenetic substrates in paired tumor-normal samples across 28 cancer types. Red color indicates upregulation (positive value) in tumor samples, whereas blue color indicates downregulation (negative value) in tumor samples compared with normal samples. The dysregulation scores in the heatmap are *Z*-scaled and centered. Right-side rectangles represent ERs with different substrate types.

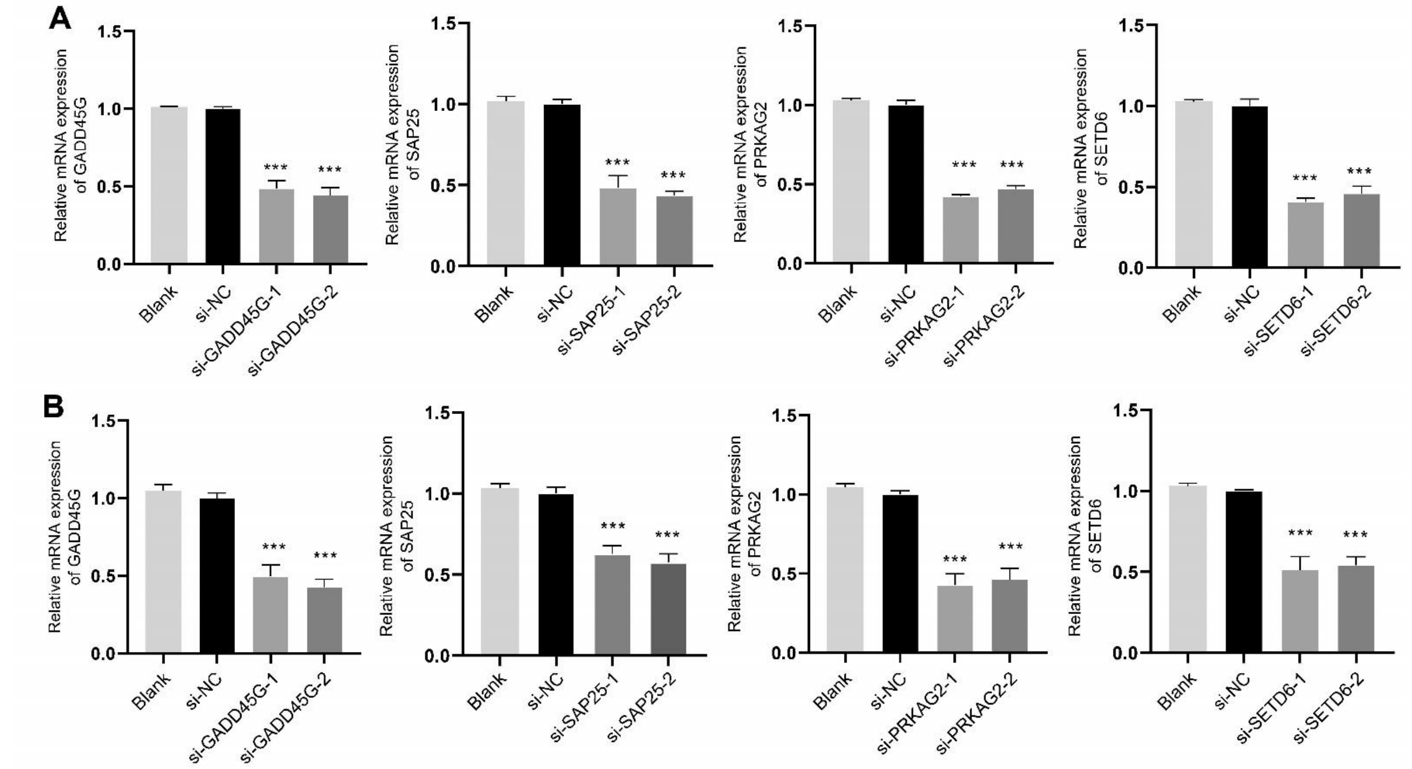

Figure S4. **Barplots of the mRNA relative expression of four candidate cancer genes in two cancer cell lines, as determined by qRT-PCR. (A)** The mRNA expression of four randomly chosen dysregulated cER genes are quantified relative to *GAPDH* in SK-mel-2 cell line ($n = 3$), **(B)** The mRNA expression of four randomly chosen dysregulated cER genes were quantified relative to *GAPDH* in A549 cell line ($n = 3$). NC, the non-targeting siRNA, was used as the negative control. An unpaired *t*-test was used to compare the si-NC and si-Gene group statistically. Significance is indicated by star symbol, *** ($P < 0.001$).

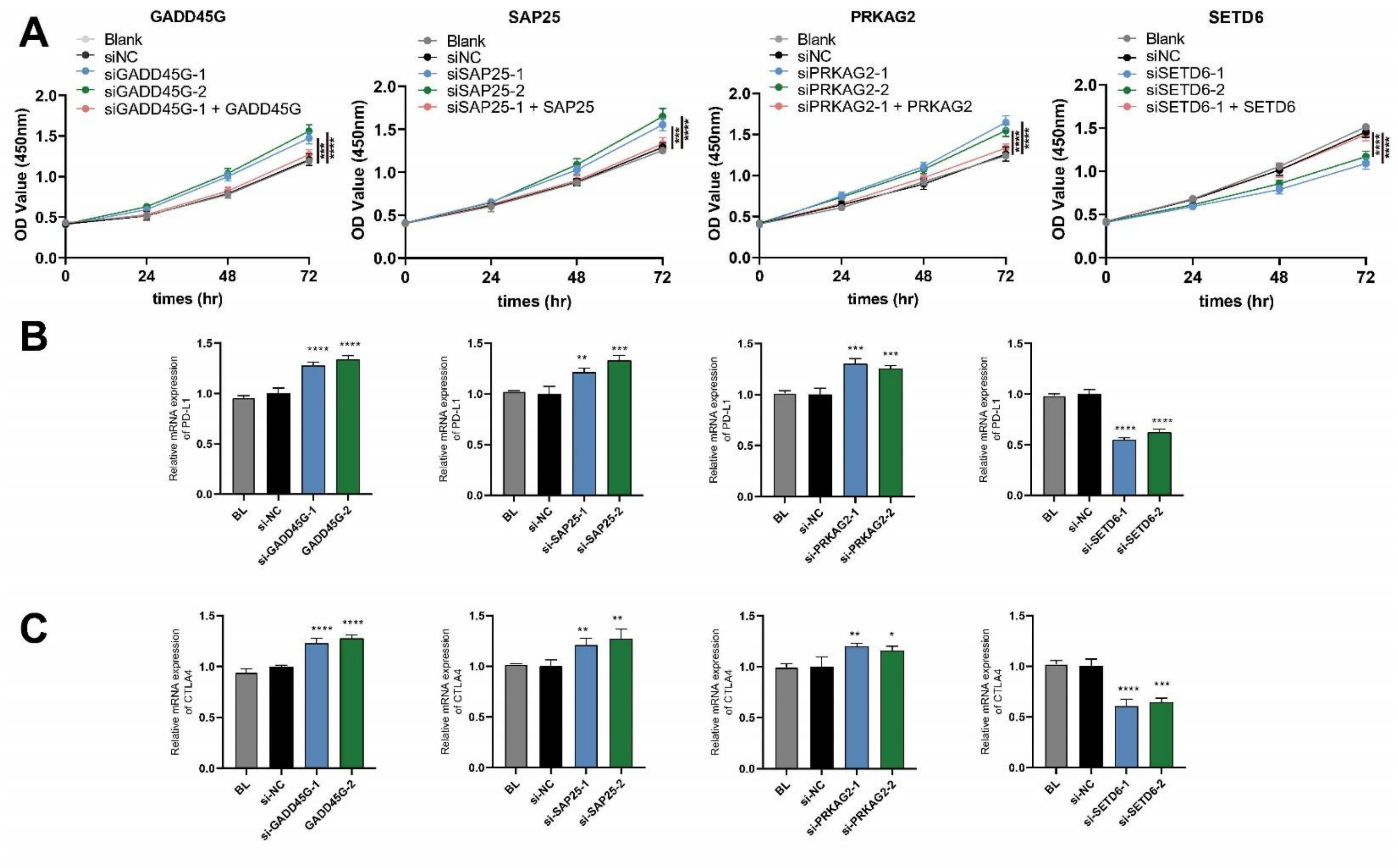

Figure S5. **Functional investigation of four candidate cER genes in A549 cells. (A)** Four randomly chosen dysregulated cER genes were investigated in A549 cell line (n = 4) by CCK-8 assays. **(B)** The PD-L1 mRNA expression of four candidate cancer genes in A549 cell lines, as determined by qRT-PCR (n = 3). **(C)** The CTLA4 mRNA expression of four candidate cancer genes in A549 cell lines, as determined by qRT-PCR (n = 3). Comparison was performed between si-NC and si-Gene. P-values are calculated by unpaired t-test and indicated by star symbol, *, $P < 0.05$; **, $P < 0.01$; ***, $P < 0.001$; ****, $P < 0.0001$.

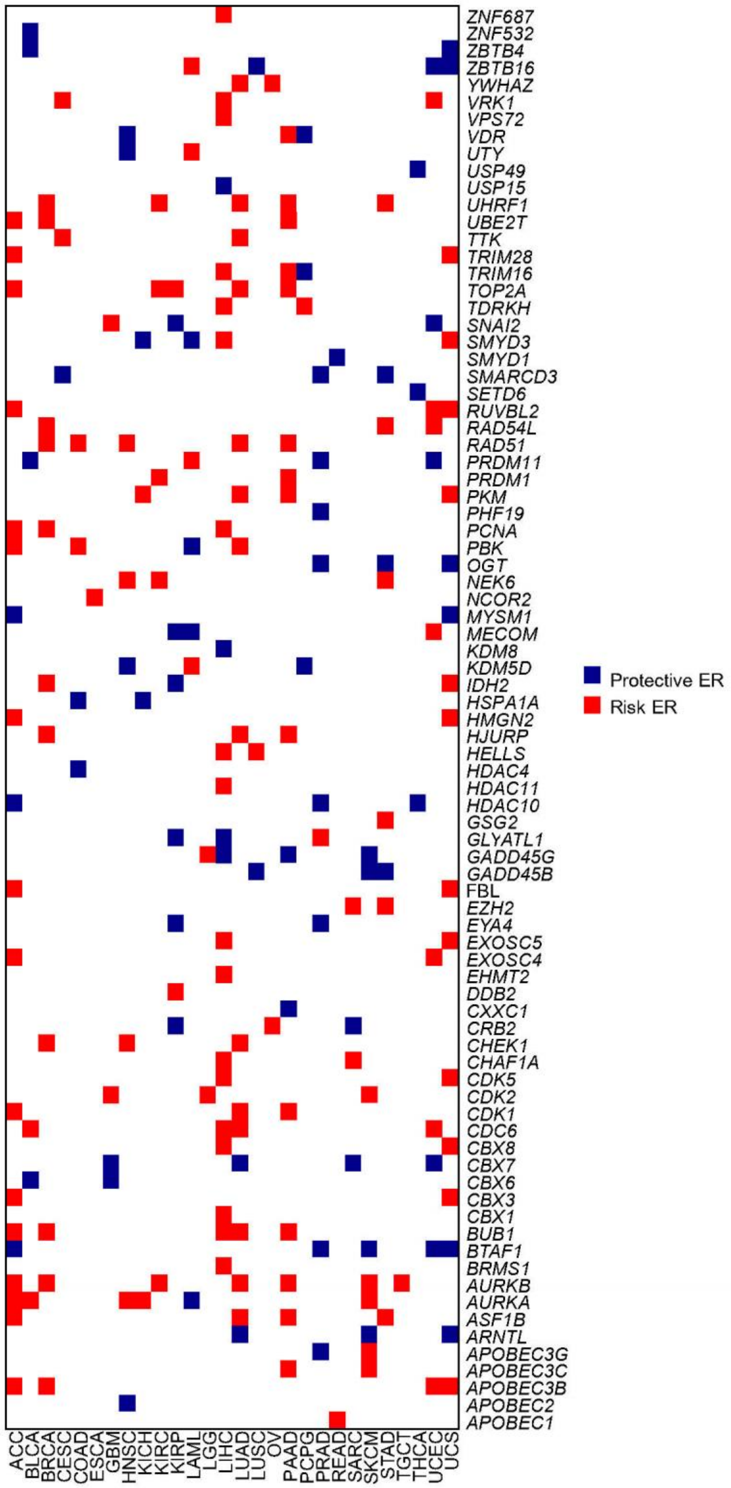

Figure S6. Heatmap of DEGs associated with overall survival in different cancer types.

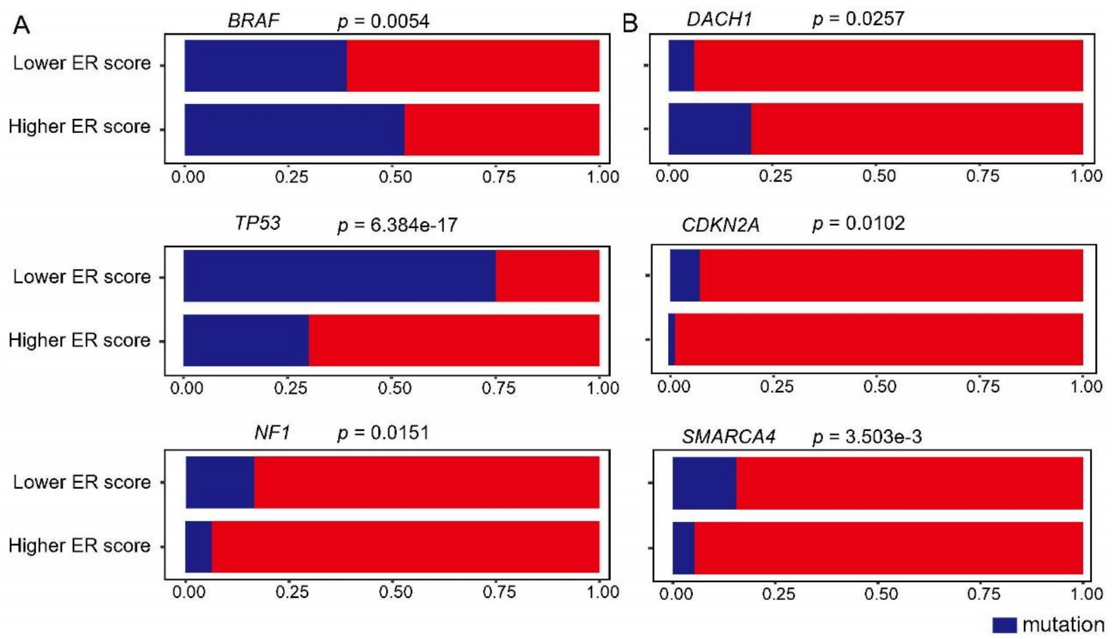

Figure S7. Comparison of the driver gene mutations between different patient groups. The distribution of selected driver genes with different mutational fractions between two signature score groups for (A) SKCM, and (B) LUAD cancer type. Depicted *p*-values are calculated by Fisher's exact tests.

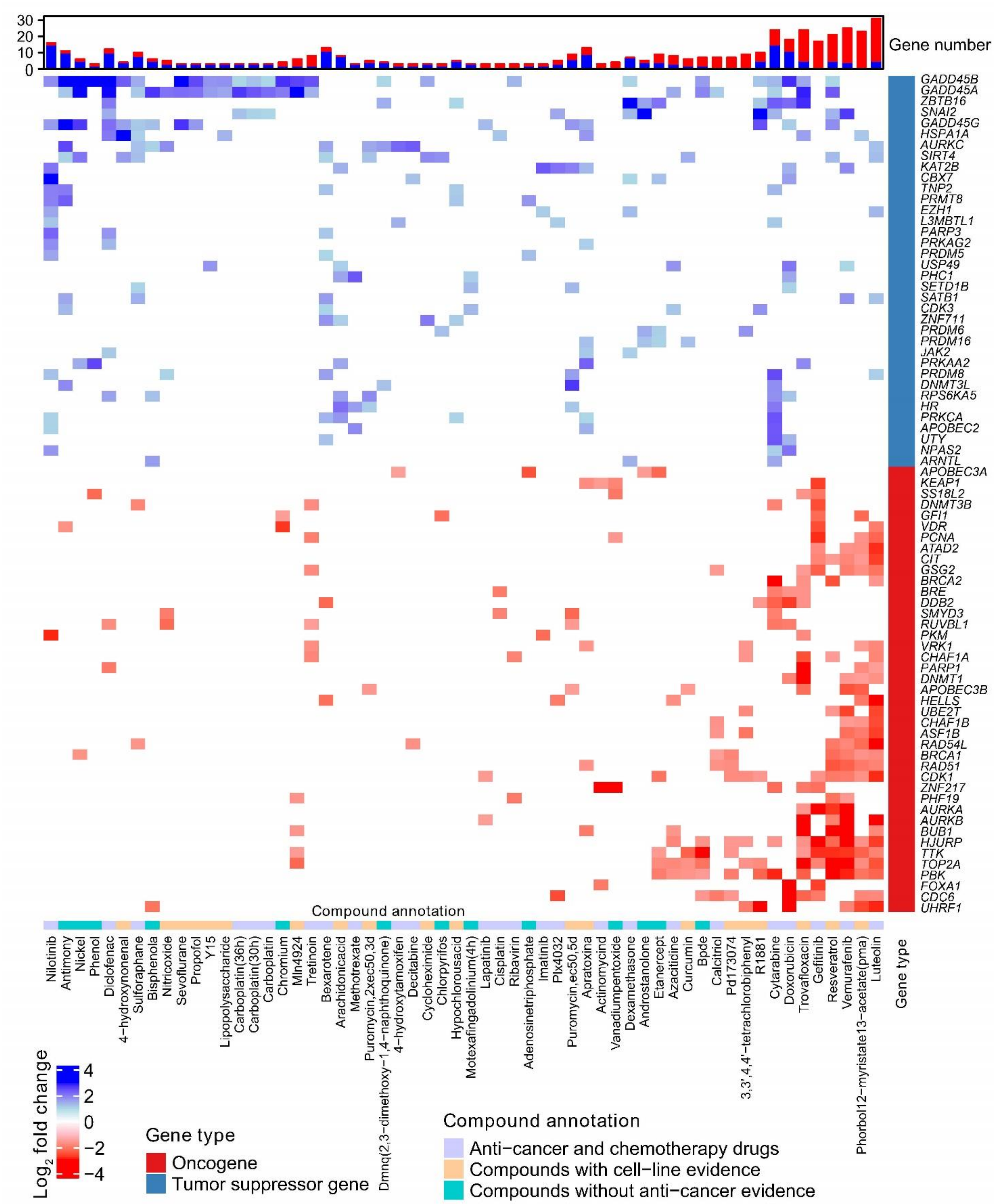

Figure S8. Heatmap of the log$_2$-transformed gene expression fold changes of the DEGs perturbed by compounds/medicines. The stacked bar plot above the heatmap indicates the total number of upregulated (blue color) or downregulated (red color) genes. DEGs, dysregulated ER genes.

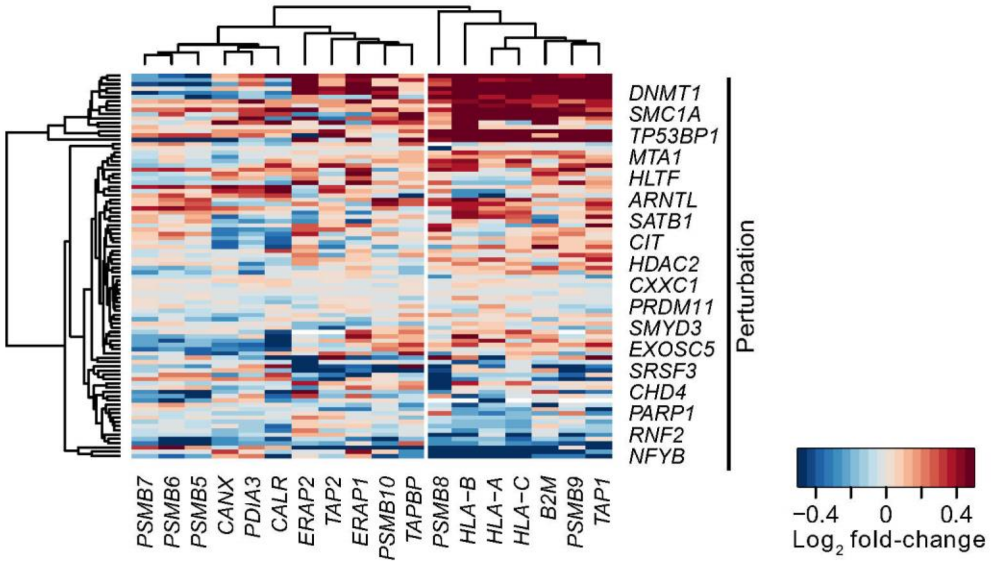

Figure S9. Perturbed ER gene expression can cause the gene expression dysregulation in the antigen presentation and processing pathway. The gene expression data is downloaded from the GPSAdb database, and the data associated only with the ER genes and the genes in the antigen presentation and processing pathway are extracted. Red color indicates upregulation upon the ER gene knockdown, whereas blue color indicates downregulation upon the ER gene knockdown.

Supplemental Tables

Table S1. The list of the human epigenetic regulator genes used in this study.
Table S2. The dysregulation scores of the significantly dysregulated epigenetic regulator genes.
Table S3. The list of the dysregulated epigenetic regulator genes that could be identified by the proposed dysregulation score method only but not log$_2$FC method.
Table S4. The gene expression responses data of the epigenetic regulator genes for different medicines or compounds.
Table S5. A summary of the qRT-PCR primers used in this study.
